# Diversity of *Escherichia coli* from Faecal Samples of Danish Calves with Diarrhoea

**DOI:** 10.3390/vetsci12100987

**Published:** 2025-10-13

**Authors:** Anna Luiza Farias Alencar, Abdurrahman Hassan Jibril, Birgitta Svensmark, Lene Agerskov, Henrik Læssøe Martin, Marc Stegger, André Becker Saidenberg, Gang Liu, Yaovi Mahuton Gildas Hounmanou, Annette Sønderholm Juel, John Elmerdahl Olsen, Rikke Heidemann Olsen

**Affiliations:** 1 Section for Bacteria and Viruses, Department for Veterinary and Animal Sciences, Faculty of Health and Medical Sciences, Frederiksberg C., 1870 Copenhagen, Denmark; alfal@aqua.dtu.dk (A.L.F.A.); gil@sund.ku.dk (Y.M.G.H.); cava@sund.ku.dk (R.H.O.); 2 Section for Fish and Shellfish Diseases, Danish Technical University Aqua, Technical University of Denmark, 2800 Kongens Lyngby, Denmark; 3 One Health Institute, Usmanu Danfodiyo University Sokoto, Sokoto 2346, Nigeria; jibril.hassan@udusok.edu.ng; 4 LVK, Cattle Practice, 9500 Hobro, Denmark; bs@lvk.dk (B.S.); lj@lvk.dk (L.A.); 5 SEGES Innovation, 8200 Aarhus, Denmark; hlm@seges.dk; 6 Department of Sequencing and Informatics, Statens Serum Institut, 2300 Copenhagen S., Denmark; mtg@ssi.dk (M.S.); adbs@ssi.dk (A.B.S.); 7 Antimicrobial Resistance and Infectious Disease Laboratory, Harry Butler Institute, Murdoch University, Murdoch, WA 6150, Australia; 8 College of Veterinary Medicine, Qingdao Agricultural University, Qingdao 266109, China; gangliu@qau.edu.cn; 9 Veterinary Laboratory, Danish Agriculture and Food Council, 8620 Kjellerup, Denmark; ansj@lf.dk

**Keywords:** calf diarrhoea, *Escherichia coli*, population diversity

## Abstract

**Simple Summary:**

Calf diarrhoea is a major health and welfare problem in dairy farming, often linked to infections with *Escherichia coli* (*E. coli*). Traditionally, strains carrying the F5 (K99) fimbriae have been considered the main cause of *E. coli*-associated diarrhoea. However, recent studies, including ours, show that this is no longer the case. In this study, we examined 391 faecal samples from Danish calves with diarrhoea and found that *E. coli* was present in most cases, but with a remarkable diversity of types both within individual calves and between calves. More than two-thirds of the samples contained multiple *E. coli* types. Surprisingly, only 4% of samples contained the classic F5 fimbriae, while a large proportion carried genes associated with other pathotypes, such as diffusely adherent *E. coli* (DAEC) and extraintestinal pathogenic *E. coli* (ExPEC). These findings suggest that calf diarrhoea in Denmark is now linked to a wide range of *E. coli* types rather than one dominant pathotype. This shift has important implications for diagnosis, treatment, and prevention, as selecting only one isolate from a sample may overlook relevant diversity, and vaccines targeting only F5-positive strains may no longer be effective.

**Abstract:**

Several different pathogens, including *Escherichia coli*, are strongly associated with calf diarrhoea. The population diversity of intestinal *E. coli* within each diarrhetic calf and between diarrhetic calves is not well understood. In the present study, 391 faecal samples were obtained during 2023–2024 from Danish dairy calves with diarrhoea. Semi-quantified growth estimates of *E. coli* after culturing did not reflect the diarrhetic grade nor whether *E. coli* was the only pathogen observed in the sample. From each sample, five isolates were subjected to multiple-locus variable-number tandem repeat analysis (MLVA) and revealed that 70% of faecal samples contained more than one type of *E. coli.* Genotyping, sequence typing and in silico serotyping showed a large diversity of *E. coli* between faecal samples. Surprisingly, isolates with a genotype representing mixed features of Diffusely adhering *E. coli*/Extraintestinal pathogenic *E. coli* were found in 25% of the isolates, while the classic Enterotoxigenic *E. coli* genotype was only observed in 5% of the isolates, and only 4% of the faecal samples were positive for *E. coli* F5 (K99) fimbriae, as determined by PCR. In conclusion, a diverse population of (non-F5) *E. coli* is associated with diarrhoea in calves. High genomic diversity of *E. coli* within samples needs to be considered when selecting only one isolate for antimicrobial resistance profiling and vaccination measurements.

## 1. Introduction

Diarrhoea is a common health problem in young calves that can lead to significant welfare problems and great economic losses to cattle producers worldwide. It is a multifactorial disease influenced by the interaction between infectious agents, management practices and environmental factors [1]. Bovine rotavirus, bovine coronavirus, bovine viral diarrhoea virus, *Salmonella* enterica, *Clostridium perfringens* (Type A, B and C), *Cryptosporidium parvum*, *Eimeria* and *Escherichia coli* are recognised as major pathogens associated with this disease [2,3]. Among these, *E. coli* is commonly recognised as one of the most important causes of diarrhoea during the neonatal period [4,5].

Good management practices focusing on hygiene and prevention of the failure of passive colostrum transfer to the calf [6,7] have been shown to play important roles in the occurrence and development of calf diarrhoea. Enterotoxigenic *E. coli* strains (ETEC) carrying the F5 (K99) fimbriae and the heat-stable enterotoxin (STa) have historically been considered the most important pathotype associated with diarrhoea in calves [4,5,8], with the K99 fimbrial adhesin recognised as an important antigen in diagnostic assays and vaccine strategies. It has, however, long been recognised that other pathotypes such as enteropathogenic (EPEC), enterohaemorrhagic (EHEC), and enteroaggregative (EAEC) *E. coli* may be found in stool samples from calves presenting diarrhoea [9,10].

Recently, a low prevalence of F5 STa *E. coli* has been reported in samples from diarrheic calves in Denmark and other countries [4,11,12,13]. One study showed that such samples may show massive growth of *E. coli* [13], without carrying out further characterisation of the strains. Since most modern diagnostics for calf diarrhoea are carried out using molecular methods, for example, the multiplex PCR as suggested by Pansri et al. (2022) [12], there is a need to determine whether such samples reflect a non-specific blooming of *E. coli*, or whether other pathotypes than ETEC F5 Sta play a role in the pathogenesis. Thus, the aim of the current study was to determine whether stool samples from calves suffering from diarrhoea contained one or more types of *E. coli*, and which pathotypes, other than F5 STa ETEC, could be identified. Addressing this gap is crucial for improving diagnostic approaches and guiding preventive strategies, including vaccine design.

## 2. Materials and Methods

### 2.1. Sampling of E. coli

A total of 391 faecal samples from Danish dairy calves with diarrhoea were obtained from 60 herds by a Danish veterinary consultant company (LVK) in the period from January 2023 to May 2024. All samples were submitted to a multiplex qPCR to detect the presence of *Clostridium perfringens* (Type A, B and C), *Salmonella* Dublin, bovine rotavirus, bovine coronavirus, *Cryptosporidium parvum*, *Coccidia* (Eimeria) and *E. coli* with fimbria F5 [12]. Each sample was scored individually by two trained technicians to be classified as diarrhetic level I, II or III, in which score I was assigned to samples presenting standard to slight decreased faecal structure (“pudding-like” consistence), score II was given to faecal samples showing a loose structure (“pancake dough-like” consistence) and score III was assigned to faecal samples demonstrating a watery consistence (+/-yellowish colour) (“orange juice”-like). In addition to the qPCR, samples were plated on SSI enterobacteria agar (SSI Diagnostica, Hillerød, Denmark) to confirm growth of *E. coli*. After incubation, the growth of *E. coli* was semi-quantitatively defined as massive (pure culture, full plate), moderate (between 30 and 60% of colonies are tentative *E. coli*), weak (>30% colonies are tentative *E. coli*) or negative for *E. coli* by visual inspection of agar plates. Samples negative for *E. coli* F5 in qPCR but with moderate to massive growth of *E. coli* on the SSI enterobacteria agar were shipped to Veterinary Clinical Microbiology (VCM) at the University of Copenhagen. From each sample, five isolates of *E. coli* were sub-cultured onto blood agar plates (Oxoid, Blood agar base III, Fischer Scientific, Roskilde, Denmark with 5% sterile bovine blood), incubated for 24 h at 37 °C, and each isolate was subsequently stored in Müller–Hinton broth (Fisher Scientific, Roskilde, Denmark) supplemented with 15% glycerol at −80 °C until further characterisation.

In addition, a total of 49 diarrhetic faecal samples were obtained from five Danish dairy farms (9–10 sampled calves per farm) with a previous history of *E. coli* calf diarrhoea (field samples). Faecal samples were shipped on ice to the University of Copenhagen. Ten microliters from each faecal sample were plated on MacConkey agar (Oxoid, Fisher Scientific, Roskilde Denmark) and incubated for 24 h. From MacConkey agar, five colonies with *E. coli* characteristics (bright pink colonies) were sub-cultured on blood agar and incubated for 24 h at 37 °C. Each isolate was verified as *E. coli* by PCR according to Chen & Griffiths (1998) [14]. All isolated *E. coli* were stored in Müller–Hinton (Fisher Scientific, Roskilde, Denmark) supplemented with 15% glycerol at −80 °C until further characterisation.

Furthermore, to extend the sample collection, 22 additional isolates of *E. coli* were obtained from clinical cases of presumed *E. coli* diarrhoea in Danish calves (field isolates) submitted to the National Livestock laboratory in Kjellerup, Denmark, and six isolates obtained as part of a published study (Herrero Fresno et al., 2023) [11] were included in the present study. These isolates were all obtained from 2022 to 2024 and originated from 25 farms.

The study was approved by the Animal Ethics Institutional Review Board, Department of Veterinary and Animal Sciences, University of Copenhagen (AEIRB number 2024-01-VCM-001A).

### 2.2. Multiple-Locus Variable-Number Tandem Repeat Analysis (MLVA)

To investigate the *E. coli* diversity within each faecal sample, a subset of samples (*n* = 35) was submitted for MLVA. For this analysis, five *E. coli* isolates randomly picked from the primary plate of each diarrhetic sample were included. For comparison, two *E. coli* F5 positive samples (determined by qPCR) were also submitted to MLVA (five isolates from each sample). Boiling lysate of each isolate was used as DNA template, while primers, agarose gel and PCR conditions were set as previously reported by Caméléna et al. (2019) [15].

### 2.3. Selection of Strains for Whole Genome Sequencing (WGS)

Based on the MLVA results, 1–5 isolates per sample were selected for WGS-based analyses. This included one isolate of the most prevalent MLVA band profile(s) out of each sample (containing up to five MLVA-types per sample). Ninety-nine isolates were subjected to WGS based on these selection criteria. Further, 34 non-MLVA typed *E. coli* isolates from the veterinary practice (LVK) and 28 non-MLVA typed *E. coli* isolates from the diagnostic laboratory of the livestock industry were submitted for WGS.

### 2.4. DNA Purification and WGS

WGS was carried out following two protocols. In the first, which was used for WGS analyses of strains selected based on the MLVA analyses, Genomic DNA was extracted utilising an enzymatic pre-lysis step (Phosphate-buffered Saline (pH 7.2 diluted to 1× in nuclease free water (Fisher Scientific, Roskilde, Denmark), 20 mM Tris-HCl (pH 8) (Fisher Scientific, Roskilde, Denmark), 2 mM EDTA (Fisher Scientific, Roskilde, Denmark), 1.2% Triton X-100 (Merch, Fisher Scientific, Roskilde, Denmark), 20 mg/mL Lysozyme (Sigma, Fischer Scientific, Roskilde, Denmark), and 1.7 mg/mL Proteinase K (Roche, Copenhagen, Denmark), prior to automated purification using the MagNA Pure 96 DNA and Viral NA Small Volume Kit and DNA Blood ds SV 2.0 protocol (Roche Diagnostics, Copenhagen, Denmark). Quantification was performed using the Qubit fluorometer (Invitrogen, Waltham, MA, USA). Subsequently, libraries were constructed, and WGS was performed utilising the Nextera XT Kit (Illumina, Little Chesterford, UK) and 300-cycle kits on the NextSeq 550 (Illumina, Little Chesterford, UK) platform according to the manufacturer’s instructions. Quality control of the obtained sequencing data was conducted using Bifrost (https://github.com/ssi-dk/bifrost, accessed January to May 2025) to ensure adequate sequencing depth (minimum average of 25 × coverage) and species verification, and identify contamination issues.

For *E. coli* strains obtained from clinical practice and the livestock diagnostic laboratory, one single colony of each *E. coli* isolate was picked from blood agar plates and added to 10 mL of Luria–Bertani (LB) broth (Oxoid, Fisher Scientific, Roskilde, Denmark), which was incubated overnight at 37 °C with shaking at 125 RPM. The cultures were homogenised by vortexing, and 3 mL (split in two) was used for DNA purification with the DNeasy Blood & Tissue kit (Qiagen GmbH, Hilden, Germany) and treated with RNAse A according to the manufacturer’s instructions. The DNA was eluted in 50 µL of buffer AE and assessed for quality and concentration using Nanodrop (inclusion criteria A260:280 > 1.80 and concentration higher than 50 ng/µL) and in an agarose gel (1%). The DNA was stored at −20 °C until whole genome sequencing by Miseq Illumina (NGS-MiSeq) (Illumina, Little Chesterford, UK) using Illumina DNA Prep and Miseq reagent kit V3. (Illumina, Little Chesterford, UK ). Assessment of raw reads was performed by FastQC version v0.11.9 (http://www.bioinformatics.babraham.ac.uk/projects/fastqc, accessed September 2024 to March 2025). Sequencing coverage cut-off was 29×. Trimommatic v.0.39 (Bolger, Lohse, and Usadel 2014) was used to trim lower-quality reads and remove adapters.

### 2.5. De Novo Assembly and Typing

De novo assemblies were generated with both read pairs using SPAdes v4.0 [16] and QUAST v5.3 [17] was used for assessing assembly quality. Prediction of serotypes was performed in silico on assemblies utilising SerotypeFinder, 2.0 [18], sequence types (ST) were identified using MLST 2.0 [19], virulence genes were identified using Virulence Finder 2.0 [20].

### 2.6. Pathotype Prediction

Pathotypes of the sequenced isolates were predicted based on the presence of virulence genes according to published suggestions. For the enteropathogenic *E. coli* (ETEC, STEC, EPEC, enteroaggregative (EAEC) and diffusely adhering (DAEC) *E. coli*), definitions were based on Geurtsen et al. 2022 [21] and Pakbin (2021) [22]. For extraintestinal pathogenic (ExPEC) *E. coli* (general ExPEC, avian pathogenic (APEC) and uropathogenic (UPEC) *E. coli*), definitions were according to those previously defined by others [23,24]. In addition to these few signature genes, additional genes were added where deemed necessary, as detailed in Table S1. The final classification, including classification into hybrid types, was performed based on the full list of virulence genes.

### 2.7. Statistical Analysis

A chi-square test was applied using GraphPad Prism Version 9.3.1 for Windows (GraphPad Software, San Diego, CA, USA) to assess if groups (grouped by diarrhetic score) differed statistically. The value of *p* ≤ 0.05 was considered statistically significant.

## 3. Results

### 3.1. E. coli-Positive Faecal Samples and Diarrhetic Score

**Figure 1:**
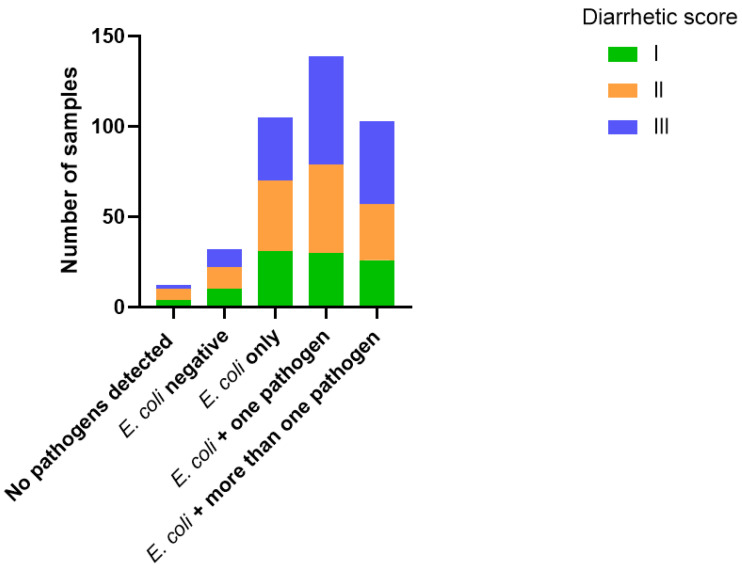
Distribution of diarrhetic scores in faecal samples obtained from Danish calves with diarrhoea. A total of 391 faecal samples were assigned a diarrhetic score (I-III) and assessed for the presence of nine diarrhoea-associated pathogens, including *E. coli*. Detection of the pathogens was based on qPCR results for all pathogens and also culturing for *E. coli*).

Initially, a total of 391 faecal samples obtained from diarrhetic calves were assessed for the presence of *E. coli*, either through qPCR (detecting only F5-positive *E. coli*) or by culturing methods, detecting the presence of *E. coli* (F5-independent); additional qPCR was conducted to detect eight other diarrhoea-associated pathogens (as presented under the “Materials and Methods” Section). In 12/391 of the samples, none of the investigated pathogens were detected, and no growth of *E. coli* was observed. In the remaining samples, at least one pathogen was detected. *E. coli* was detected in 362/391 (92%) of the samples, of which 14/362 samples (4%) were positive for the F5 fimbriae. Most samples (151/391, 38%) were assigned diarrhetic score III, while 25% (101/391) and 35% (137/391) of the samples were assigned diarrhetic scores of I and II, respectively. The distribution of diarrhetic scores I, II or III did not differ significantly between the group of samples in which *E. coli* was the only detected pathogen (n = 105), the group of samples in which *E. coli* was present together with one additional detected pathogen (n = 139) and the group where *E. coli* was present together with two or more detected pathogens (n = 136) (*p* = 0.416) (Figure 1).

**Figure 2:**
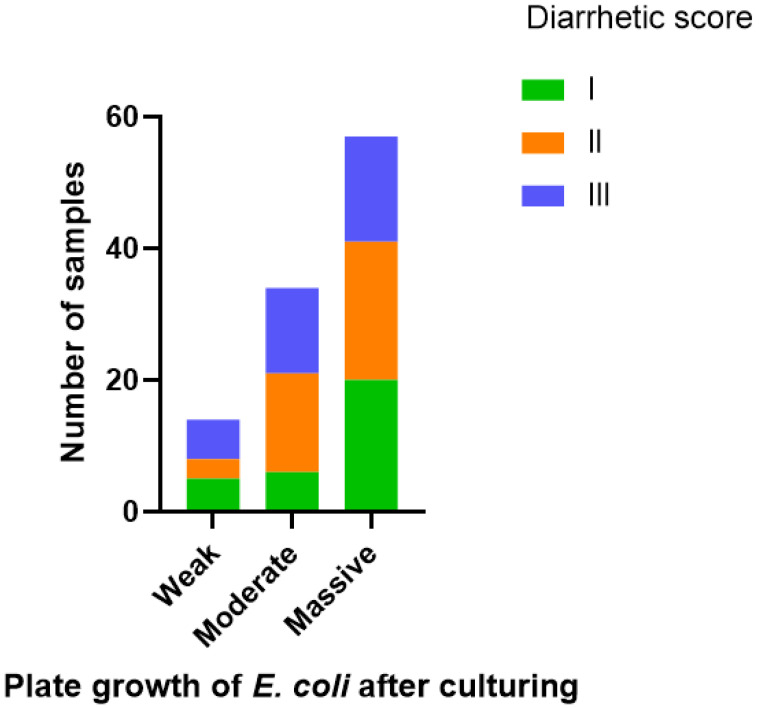
Distribution of diarrhetic scores I, II or III in faecal samples in which *E. coli* was the only detected pathogen (105 samples). Samples were grouped based on the quantity of *E. coli* growth (weak, massive or moderate).

The amounts of *E. coli* were semi-quantified based on the culturing results as *E. coli* negative, weak growth, moderate growth or massive growth. The rates of diarrhetic scores I, II or III in the group in which *E. coli* was the only detected pathogen were not significantly different depending on whether *E. coli* growth was rated as weak, moderate or massive (*p* = 0.299) (Figure 2).

**Figure 3:**
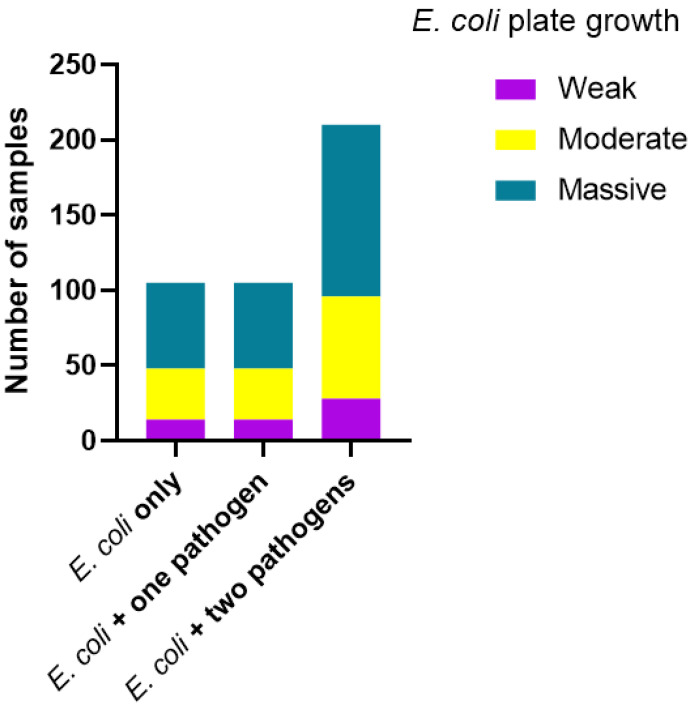
Distribution of plates with weak, moderate or massive growth of *E. coli* after culturing of 255 faecal samples from calves with diarrhoea. The samples were grouped according to whether *E. coli* was the only detected pathogen in the sample or at least one other pathogen was detected in the sample. There were no significant differences between the groups (*p* = 0.999).

The quantity of *E. coli* tended to increase when two or more other pathogens were present in the samples (plate growth) (Figure 3); however, the difference between groups was not statistically significant (*p* = 0.999).

### 3.2. E. coli MLVA Typing and Diversity

A total of 455 isolates from 91 samples (five isolates per sample) were chosen to be characterised by MLVA. Each isolate was assigned to an MLVA pattern based on band patterns, and samples were assigned an MLVA type depending on the number of different MLVA patterns observed in the five strains, which were typed from the same sample. In MLVA type 1, all *E. coli* isolates in the sample had the same band pattern; in MLVA type 2, four out of five *E. coli* isolates had the same band pattern; in MLVA type 3, three out of five *E. coli* isolates had the sample had different band patterns; in MLVA type 4, four out of five *E. coli* isolates had different band patterns; and in MLVA type 5, all five *E. coli* isolates had different band patterns. MLVA type 1 was the most common type (31% of samples), closely followed by MLVA type 4 (27% of samples) (Table 1). An MLVA was also carried out on strains obtained from presumed clinical cases. For these samples, no information was available on other pathogens present in the samples. The distribution of MLVA types did not differ markedly from the distribution above, with the exception that no samples with MLVA type 5 were present (Table 1).

### 3.3. MLVA Types, Diarrhetic Scores and Quantity of E. coli

**Figure 4:**
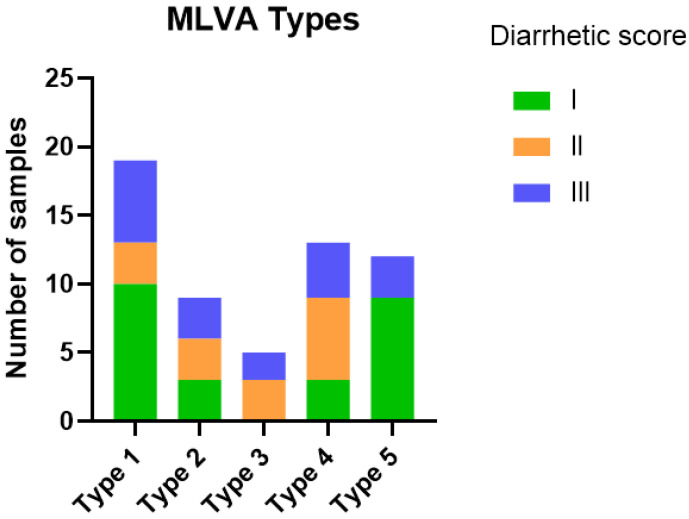
MLVA types vs. diarrhetic scores. Distribution of MLVA types among 58 faecal samples with diarrhetic scores I to III. Five isolates per sample were included in the analysis. MLVA type 1: Band patterns identical for all five isolates; MLVA type 2: Band patterns identical for 4/5 isolates; MLVA type 3: Band patterns identical for 3/5 isolates; MLVA type 4: Band patterns identical for 2/5 isolates; MLVA 5: Band patterns different for all five isolates. No significant difference was observed between groups (*p* = 0.510).

**Figure 5:**
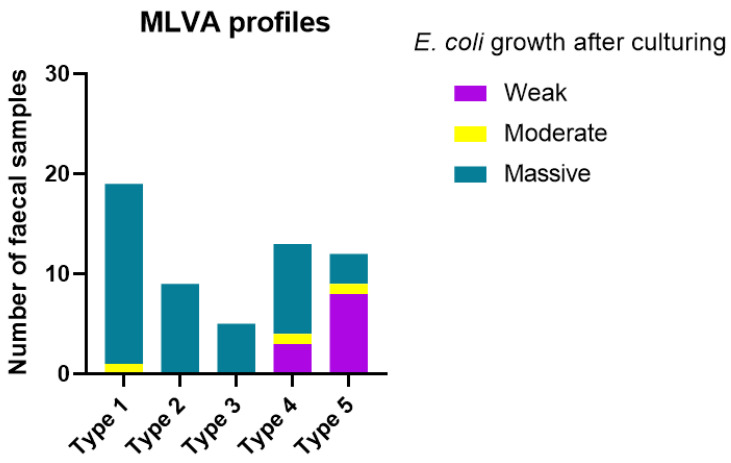
MLVA type and semi-quantitative estimation of *E. coli*. Distribution of MLVA types among 58 faecal samples divided into groups based on the culturing results of *E. coli* (categorised as weak, moderate or massive growth). Five isolates per sample were included in the analysis. MLVA type 1: Band patterns identical for all five isolates; MLVA type 2: Band patterns identical for 4/5 isolates; MLVA type 3: Band patterns identical for 3/5 isolates; MLVA type 4: Band patterns identical for 2/5 isolates; MLVA 5: Band patterns different for all five isolates. No significant difference was observed between groups (*p* = 0.416).

In 58 faecal samples, we had information that allowed us to group the samples based on MLVA types and diarrhetic score (Figure 4) and quantity of *E. coli* (Figure 5). There was no association (*p* = 0.510) between MLVA type and the diarrhetic score of the sample (Figure 4), e.g., samples with diarrhetic score I constituted the majority of the samples for both the MLVA type 1 group and MLVA type 5 group.

In contrast, there was a more marked difference between samples with different MLVA types when grouped according to plate growth of *E. coli* (Figure 5), e.g., 18/19 (95%) of the samples with MLVA type 1 demonstrated massive growth, whereas only 3/12 (25%) of the samples with MLVA type 5 demonstrated massive growth, and most of the samples in this group (8/12, 67%) demonstrated only weak growth.

### 3.4. E. coli Genotypes, Sequencetypes, and Serotypes

A total of 161 *E. coli* isolates were subject to WGS (see Supplementary Table S3 for selection of strains for WGS) and annotated to identify pathotype, sequence types (ST) and serotypes. In total, 71/161 (44%) of the strains could not be assigned to a pathotype based on the annotated virulence genes (Table 2). These strains were further analysed to determine if they were most probably commensal *E. coli;* however, known virulence genes were detected in all strains (Supplementary Table S3). Among the known pathotypes, Diffusely Adherent *E. coli* (DAEC) was the most frequently assigned genotype, found in 55/161 (34%) of the isolates. Of these, 45 were of a mixed pathotype, most commonly DAEC/ExPEC (41/55). Other pathotypes detected consisted of ETEC, EHEC, EPEC and ExPEC, some of which were classified to be APEC.

The isolates were found to belong to 41 different STs, of which ST10, ST58, ST69 and ST88 were the most frequently observed, assigned to 11%, 11%, 9% and 8% of strains, respectively (Supplementary Table S4). Approximately 20% (29/161) of the STs observed were represented by two or fewer isolates. A large diversity of serotypes was observed, and even the most frequently observed type (O101:H9) was only observed among 16/161 (4%) of the isolates (Supplementary Table S5).

## 4. Discussion

The present study aimed to determine whether quantity, diversity of *E. coli* strains and pathotype of strains differed in stool samples from diarrhetic calves less than one month of age, depending on whether *E. coli* was the only pathogen isolated or whether other known causative agents of calf diarrhoea were detected.

Culturing of the faecal samples showed growth of *E. coli* in 87% of all stool samples. Only 4% of the samples were positive for the F5 fimbriae by PCR, which is indicative of ETEC presence. The F5 STa ETEC has previously been reported as a common diarrhetic *E. coli* type in Danish calves [25,26] and elsewhere [8]. The low prevalence observed here suggests that a shift in the types of diarrhetic *E. coli* has occurred. Our findings are in agreement with previous studies performed in Denmark [11,12,13], Canada [8], Norway [27] and Uruguay [28]. On the other hand, higher prevalences have been reported by Coskun & Sahin [5] and in previous studies from France [29] and Iran [30]. The reason for this marked difference in ETEC F5 STa occurrence may be related to different management systems and different genetics of animals in different countries. Further studies are needed to determine this.

In 105 samples, *E. coli* was the only detected pathogen and therefore it was assumed to be the causative agent of the diarrhoea. Such samples did not show a uniform severity of diarrhoea, as samples with diarrhetic scores I, II and III were similarly distributed and did not differ according to the extent of *E. coli* growth. Thus, it is not possible to use consistency of the faecal material as an indicator of *E. coli* diarrhoea. Possible reasons are that different pathotypes of *E. coli* cause different degrees of diarrhoea, and that different pathotypes were present in the same sample or that the degree of diarrhoea was related to the quantity of *E. coli* present. We therefore went on to investigate these factors.

We found no correlation between the semi-quantitative measure of *E. coli* in the sample and the diarrhetic score, e.g., fewer samples with massive growth of *E. coli* came from faecal samples with diarrhetic score III than from samples with diarrhetic score I. Again, this may simply be due to a difference in pathotype or the number of different strains present, which will be discussed later. When other pathogens were detected in the same faecal sample together with *E. coli*, most samples would have a massive growth of *E. coli*, which could indicate that the presence of other pathogens could lead to an *E. coli* dysbiosis/overgrowth, as previously observed in humans [31]. Further studies, including metagenomic studies, are needed to confirm this.

Traditionally, when diagnosing *E. coli*-associated calf diarrhoea, only one colony per cultured faecal/intestinal sample is characterised [32,33,34,35]. This is performed under the assumption that the population is homogeneous, because the strains causing the diarrhoea will have outgrown other types. However, MLVA typing results of the current study showed that in 70% of the samples at least two different types of *E. coli* could be detected among the five isolates analysed per sample, and for 13% of the samples each of the five isolates had a different MLVA profile. Faecal samples with only one MLVA pattern were more likely than the other types to show massive growth of *E. coli*, showing that there is an element of truth in the above assumption of overgrowth with one strain happening in such samples. However, the severity of diarrhoea did not depend on how many *E. coli* types were present in the sample. For example, the group of samples with the same MLVA pattern for all five isolates and the group of samples where all isolates were different from the other isolates regarding MLVA pattern had the same proportions of samples with diarrhetic score III. This contributes to the above conclusion that the severity of diarrhoea is not a good indicator of *E. coli*-associated diarrhoea. Indeed, genome sequencing of isolates from the different MLVA types showed that pathotypes, sequence types and serotypes did not vary systematically depending on whether the strains originated from a sample with 1, 2, 3, 4 or 5 different MLVA patterns.

From the observation above, it was clear that a more homogenous population of *E. coli* was not found in those samples from which a known pathogenic type of *E. coli* was found, compared to other samples where the *E. coli* type(s) did not belong to any known pathotype. For example, isolates of serotype O101, which have previously been reported to be associated with calf diarrhoea [36,37], was isolated from samples with all types and numbers of MLVA patterns, and ST69 strains [38], which have also previously been reported to be associated with calf diarrhoea, were exclusively found in samples with four or five different MLVA patterns. This finding has diagnostic implications if only a single colony is picked from each sample, as the type isolated could be unrelated to the diarrhoea. This also holds true when the isolate is used for an antibiogram, which will serve as a guide to the treatment.

A large diversity within STs and serotypes of *E. coli* was observed among the sequenced strains; however, the most dominating types, such as ST10, ST58, ST69 and ST88, and serotypes, such as O101 and O15, are well-known types that are associated with calf-diarrhoea [36,38,39]. Almost 20% (29/161) of the isolates had STs occurring only once or twice among the sequenced isolates, and this included several types which have not previously been reported from diarrhoea. It is likely that these rare types are commensals merely co-occurring in samples with more pathogenic types, underlining that a more thorough understanding of the diversity of *E. coli* within each faecal sample is needed.

Surprisingly, only 5% of the sequenced isolates demonstrated a classic ETEC genotype. The largest group of isolates (44%) could not be assigned to a defined pathotype based on their cargo of virulence genes. Such “no-pathotype” isolates could represent commensals co-occurring in samples with more pathogenic types, as 17% of the NG isolates did originate from faecal samples of diarrhoea score III. None of these strains were devoid of known virulence factors, and some of them carried many virulence genes, mainly of genes associated with ExPEC strains, just not enough to fulfil the combinations required to fall into a particular group in the current study. This highlights that the distinction between commensal and pathogenic *E. coli* is not easy. In common with the isolates, which could be allocated into known pathotypes, almost all these strains were shown to carry the *yehAD* gene cluster. This operon encodes a fimbriae, which has previously been shown to be important for the way EAEC strains stimulate immune responses in the intestine [40], and for robust colonisation with EHEC strains [41]. The widespread occurrence in the current study may suggest that this fimbriae type gives an advantage in the colonisation of calves, whether the strains are pathogenic or not. Further studies are needed to determine this.

The strains which could be classified into a known pathotype were mostly of DAEC or the DAEC/ExPEC hybrid type, but other types such as EHEC, EPEC and ExPEC, including APEC, were also detected. We consider the hybrid-type DAEC/ExPEC as an artefact of the definitions used in the current study, since *afa* genes (the adhesion genes of DAEC [42]) were part of the required genes in both groups. The DAEC type has previously been associated with diarrhoea [43]. Importantly, the current study can only indicate the presence of this type, as its real status as DAEC can only be detected using cell culture [44].

In contrast to DAEC, the high occurrence of ExPEC strains in the current study is surprising. In calves, ExPEC strains have been associated with umbilical infections and meningitis, and only more recently, a study of an outbreak of neonatal calf diarrhoea and pneumonia reported a multidrug-resistant ExPEC as the cause of severe mortality in calf herds in China [45]. Further studies are necessary to determine whether such strains are causative in relation to the diarrhoea, or whether they simply grow better in the inflamed bovine intestine, as suggested by studies performed in other species [46,47,48,49]. Some of the ExPEC strains were hybrid types with known diarrhoeagenic types, such as EHEC. These hybrid types of *E. coli* deserve future attention, as such types have previously been described as causing more severe disease, at least in humans [50,51].

In conclusion, the ETEC F5 type, which was previously the dominant type in the *E. coli* population related to calf diarrhoea [6,26], was found to be rare, which may indicate a shift in pathotypes circulating in diarrheic calves. Such a shift may be caused by the use of vaccines specifically targeting F5 ETEC, and it can have implications for future vaccine strategies. Large diversity was observed in the types of *E. coli* that could be isolated from calves with diarrhoea, and often one defining diarrhetic pathotype could not be detected. The large diversity is important to consider when deciding on prophylactic measurements, such a flock vaccination. It may also need to be reconsidered if a single isolate from a sample is sufficient for characterising the *E. coli* strain causing diarrhoea in terms of both pathotype and important phenotypic characteristics, such as antimicrobial sensitivity.

## Figures and Tables

**Table 1:** MLVA profiling of *E. coli* from calves with diarrhoea ^1^.

MLVA Type	N (% of All Samples)	No Other Pathogens Detected (n = 20)	With Other Pathogen (s) (n = 38)	Clinical Isolates (33)
1	28 (31%)	4	15	9
2	13 (14%)	4	5	4
3	13 (14%)	2	3	8
4	25 (27%)	9	4	12
5	12 (13%)	1	11	0

A total of 91 faecal samples were MLVA types, of which 58 of the samples were assessed for the presence of other diarrhoea-associated pathogens than *E. coli*, while the remaining 33 samples were clinical isolates from calves with presumed *E. coli* diarrhoea. The MLVA profile of each faecal sample was used to assign an MLVA type for the faecal sample. One isolate per sample with MLVA type 1 was subsequently sequenced and used for the genomic analysis (assigning of genotypes, serotypes and sequence types (ST)), while for faecal samples with MLVA type 2, 3, 4 and 5, two, three, four and five isolates, respectively, were used for genotypic characterisation. ^1^ Information on which pathotypes, sequence types and serotypes were detected in each MLVA type can be found in Supplementary Table S2.

**Table 2:** Pathotype of 161 sequenced isolates of *E. coli* obtained from calves with diarrhoea and depending on the presence of other pathogens.

	With no Other Pathogens Detected (n = 30)	With Other Pathogen(s) Detected (n = 69)	Unknown Presence of Other Pathogen(s) (Clinical Cases) (n = 62)
ETEC	0	2	6
F5 positive, tox	0	2	0
EHEC (+DAEC/ExPEC)	0	2	8
EPEC	0	1	0
DAEC	2	3	3
DAEC/EHEC	1	1	2
DAEC/ExPEC	6	21	14
ExPEC	4	5	7
NG	17	32	22

For 99 of the isolates, the faecal samples were assessed for the presence of diarrhoea-associated pathogens other than *E. coli*, while 62 isolates originated from calves with presumed *E. coli* diarrhoea (without investigation of the potential simultaneous presence of other pathogens). Based on the presence or absence of a set of defined virulence genes, each isolate was assigned a pathotype as follows: No grouping possible (NG). ETEC: Enterotoxigenic *E. coli*; EHEC: Enterohemorrhagic *E. coli*; DAEC: Diffusely Adherent *E. coli*; ExPEC: Extraintestinal Pathogenic *E. coli*. Brackets indicate that the isolate has the majority of genes to be assigned the genotype.

## Data Availability

The original contributions presented in this study are included in the article/Supplementary Materials. Further inquiries can be directed to the corresponding author(s). Sequence data for isolates EC1 to EC91 (Supplementary Table S3) were uploaded to Enterobase and are available at https://enterobase.warwick.ac.uk/species/ecoli/search_strains. Sequence data for the remaining sequenced strains are available at NCBI under SUB15644376.

## Supplementary Materials

The following supporting information can be downloaded at https://www.mdpi.com/article/10.3390/vetsci12100987/s1, Table S1: Genes used to preliminarily allocate *E. coli* strains into pathotypes; Table S2: MLVA types with information on genotypes, sequence types and serotypes observed within each MLVA type; Table S3: Metadata for each *Escherichia coli* isolate of which whole-genome sequence data is used in the paper; Table S4: Sequence typing of 161 isolates of *E. coli* obtained from calves with diarrhoea; Table S5: Serotyping of 161 isolates of *E. coli* obtained from calves with diarrhoea.

## Abbreviations

The following abbreviations are used in this manuscript:

ETEC	Enterotoxigenic *Escherichia coli*
EHEC	Enterohemorrhagic *Escherichia coli*
EPEC	Enteropathogenic *Escherichia coli*
ExPEC	Extraintestinal *Escherichia coli*
DAEC	Diffusely adhering *Escherichia coli*
APEC	Avian pathogenic *Escherichia coli*
