# Supplementary material for: Diversity of Escherichia coli from Faecal Samples of Danish Calves with Diarrhoea
Source: Vet Sci. 2025 Oct 13;12(10):987. doi: 10.3390/vetsci12100987 (PMC12568266; doi:10.3390/vetsci12100987)
Supplement: Supplementary file 1 [file vetsci-12-00987-s001.zip › Supplementary Table S1.pdf]

Supplemental Table S1. Genes used to preliminarily allocate *E. coli* strains into pathotypes[1]

Pathotype	Associated genes	Description
Enteropathogenic *E. coli* (EPEC)	*eae*	Intimin
	bfp	Bundle forming pilus
Vero (Shiga) toxin producing *E. coli*	*stx*	Shiga like toxin 1 and/or 2
Entero-haemorrhagic *E. coli* (EHEC)	Above + *ehxA*	Hemolysin[2]
Enterotoxigenic *E. coli* (ETEC)	*Sta, Stb*, *ent1*	Heat stable and/or heat labile enterotoxin
	*fanA (*F5*), f41, clpG (*CS31A*), f17*	Fimbriae[3]
Enteroaggregative *E. coli* (EAEC)	*aagR, aagA, aaf*	Aggregative adhesion systems
Diffusely adherent *E. coli* (DAEC)	*afaAD*	Adhesions
Extra intestinal pathogenic *E. coli* (general ExPEC	*papAH, sfa/focDE, afa/draBC, kpsM II and iutA*[4]	≥2 of these five markers satisfies the ExPEC status
ExPEC - avian pathogenic *E. coli* (APEC)	*iutA, iroN, iss, hlyF, ompT*[5]	≥4 of these five markers satisfies the APEC status
ExPEC – uropathogenic *E. coli* (UPEC)	*chuA, fyuA, vat and yfcV*[6]	≥3 of these four markers satisfies the UPEC status

[1]For enteropathogenic *E. coli* (ETEC, STEC, EPEC, enteroaggregative (EAEC) and diffusely adhering (DAEC) *E. coli*), definitions were based on Geurtsen *et al*. (2022) and Pakbin (2021). [2]Included as a signature gene based on Pakbin (2021); [3]Calf relevant adhesion factors selected based on Umpierrez (2021); [4]ExPEC definition according to Johnson *et al.* (2003); [5]APEC definition according to Johnson et al. (2008); [6]UPEC definition according to Spurbeck et al. (2021).
