# Supplementary material for: Diversity of Escherichia coli from Faecal Samples of Danish Calves with Diarrhoea
Source: Vet Sci. 2025 Oct 13;12(10):987. doi: 10.3390/vetsci12100987 (PMC12568266; doi:10.3390/vetsci12100987)
Supplement: Supplementary file 1 [file vetsci-12-00987-s001.zip › Supplementary Table S2. MLVA Types.pdf]

Supplemental Table S2. MLVA types with information on genotypes, Sequence types and serotypes observed within each MLVA type.

MLVA Type	N (% of all samples)	No other pathogens (n=20)	With other pathogen(s) (n=38)	Clinical isolates[2] (33)	Genotype	ST[3]	Serotype
1	28 (31%)	4	15	9	DAEC/ExPEC (n=10) NG (n=8) EHEC (n=4) ExPEC (n=2) DAEC/ EHEC (n=2) DAEC, DAEC/ExPEC	10 (n=4), 17, 21, 29, 58, 69 (n=5), 108 (n=2), 117, 167,174, 187, 219, 351, 362 (n=2), 641, 1049, 1725, 2522, 5911	0154:H30, 017/077:H18, 08:H25, H30, O101:H10 (n=2), O101:H9 (n=3), O103:H2 (n=2), O110:H2, O117:H10, O121:H10, O132:H18, O15 (n=2), O15:H18, O153, O17/O44:H18, O176:H30, O18ac:H17, O25:H8, O26:H11 (n=2), O26:H4, O33:H4, O8:H21
2	13 (14%)	4	5	4	NG (n=5), EHEC (n=2) DAEC/ ExPEC,(n=2) DAEC/ EHEC/ ExPEC	10, 32, 58, 69, 117, 155 (n=3), 219, 362, 939, 1485, 5177	H21 (n=2), H23, H51, O101:H10, O145, O17/O44:H18, O21:H9, O26:H11, O33:H4, O88:H25, O9:H25, O99a:H25
3	13 (14%)	2	3	8	NG (n=6) DAEC/ExPEC (n=5), DAEC ExPEC	58 (n=3), 69 (n=4), 88, 164, 167, 1049, 2325, 6118	H15, O10:H42, O101:H9. O132:H18, O15 (n=2), O15:H18, O154:H25,

							O160:H10, O8:H10 O8:H4, O86:H19 (n=2)
4	25 (27%)	9	4	12	NG (n=15) ExPEC (n=3) DAEC (n=2) DAEC/ExPEC (n=3), DAEC/ EHEC EHEC	10, 23, 38, 58, 69 (n=3),108, (n=5), 109,117,145, 219, 398 (n=2),410, 602, 1304, 1695, 3995, 5880, 17734	O101:H9, O107:H30, O107:H54, O108 O13/O129:H11, O13/O135:H15, O133:H20, O149, O15:H18 (n=3), O15:H4, O150:H7, O26, O28:H8, O3:H9, O32:H9, O40:H32, O52:H10, O7, O75:H7, O8:H20, O9:H21, O91:H7, O99a:H25
5	12 (13%)	1	11	0	NG (n=10) DAEC/ExPEC (n=2)	34, 38, 50, 58 (n=2), 410, 446,1485, 3381, 4701, 5236, 5756	O62:H30, O7 O150:H8, H27 (n=2), O3:H9, O88:H8, O83:H42, O50:H25, O109:H45, O45:H19, O117:H16

A total of 91 faecal samples were MLVA types, of which 58 of the samples were assessed for the presence of other diarrhoea-associated pathogens than *E. coli*, while the remaining 33 samples were clinical isolates from calves with presumed *E. coli* diarrhoea. The MLVA profile of each faecal sample was used to assign a MLVA type for the faecal sample. One isolate per samples with MLVA type 1 was subsequently sequenced and used for the genomic analysis (assigning of geno-, serotypes and sequence types (ST)), while for faecal samples with MLVA type 2,3,4 and 5, two, three, four and five isolates, respectively, were used in the for the genotypic characterization.
