# Supplementary material for: Diversity of Escherichia coli from Faecal Samples of Danish Calves with Diarrhoea
Source: Vet Sci. 2025 Oct 13;12(10):987. doi: 10.3390/vetsci12100987 (PMC12568266; doi:10.3390/vetsci12100987)
Supplement: Supplementary file 1 [file vetsci-12-00987-s001.zip › Supplementary Table S3.pdf]

Supplemental Table S3. **Metadata for each *Escherichia coli* isolate of which whole-genome sequence data is used in the paper**. LVK: Landbrugets Veterinære Konsulenttjeneste (in English: Agricultural Veterinary Advisory Service); SEGES, VetLab, GUDP: veterinary consultancies providing field fecal samples of from presumed cases of *E. coli* diarrhea; ST: Sequence type; MLVA: Multiple Locus Variable number tandem repeat Analysis (on fecal level) – if no number has been stated, the isolate has not been MLVA type; NG: No genotype could be defined; HOL: Holstien; JER: Jersey; MIX: Mixed breed; RDM: Red Danish Dairy cow; JUT: Jutland. Only samples obtained from LVK were semi-quantified regarding the extent of *E. coli* growth after culturing. qPCR analysis is performed on fecal level. If no Ct value is state, the *E. coli* isolate in question originates from a fecal sample negative for the pathogen. All samples were also analyzed by qPCR for the detection of *Clostridium perfringens Type B and C, Salmonella* Dublin and Emeria. All samples were negative for all four pathogens and therefor omitted from the table.

															**qPCR Ct value**				
**Strain Name**	**Alter-native strain name**	**Provi-der****	**ST**	**MLVA profil**	**Sero-type**	**Virulence genes**	**Patho-type**	**Farm**	**Calf study nr**	**Breed**	**Geographic placement**	**Age**	**Diarrhea grade**	**Culture *E. coli***	***Clostridium perfringens* (type A)**	***E. coli* (F5)**	***Rota-virus***	***Corona-virus***	***Cryptosporidium parvum***
EC1	100-9642-3	LVK	1485	5	O83:H42	AslA **chuA** espY2: 000868321 fdeC fimH gad hlyE **iss** kpsE **kpsMII** lpfA nlpI **ompT** terC yehB yehC yehD **yfcV**	NG	1	1	HOL, MIX	JUT	11	+	Massive	24				28
EC2	110-3591-1	LVK	10	1	O101:H10	AslA anr capU csgA gad hyE iha **iss** mcmA nlpI **papA_F19** **papA_F48** **papC** shiA terC terC tia traJ traT traT yehA yehB yehC yehD	NG	2	2	HOL, MIX	JUT	9	+++	Massive	27		21		
EC3	111-3602-1	LVK	58	1	08:H25	**afaA** **afaB** **afaC** **afaD** anr astA capU csgA espP faeI fimH gad hlyE hra iha iucC **iutA** lpfA mcmA nlpI **ompT** **papA_F19** **papA_F48** **papC** shiA sitA terC tia traJ traT yehA yehB yehC yehD	DAEC/ ExPEC	2	3	HOL, MIX	JUT	5	+	Massive	21		24		
EC4	112-3597-2	LVK	174		O121:H5	AslA air anr **chuA** cvaC eilA etsC fdeC fimH **fyuA** gad hlyE **hlyF** hra **iroN** irp2 **iss**	ExPEC (APEC)	2	4	HOL, MIX	JUT	7	+++	Massive	31		18		

						iucC **iutA**<br>kpsE<br>**kpsM_K15**<br>lpfA mchF<br>nlpI **ompT**<br>**papA_F48**<br>**papC** sitA<br>terC traT<br>tsh yehA<br>yehB yehC<br>yehD													
EC5	112535-6187-2	SEGES	69	1	O15	AslA anr<br>capU csgA<br>gad hlyE<br>hra iha **iss**<br>mcbA cbA<br>nlpI<br>**papA_F19**<br>**papA_F48**<br>**papC** terC<br>tia traJ<br>traT yehA<br>yehB yehC<br>yehD	ExPEC (APEC)	3	5	JER, MIX	FYN	<28 days							
EC6	112535-6188-1	SEGES	117	4	O150:H7	AslA anr<br>capU csgA<br>gad hlyE hra<br>iha **iss** mcmA<br>nlpI<br>**papA_F19**<br>**papA_F48**<br>**papC** terC<br>tia traJ traT<br>yehA yehB<br>yehC yehD	EHEC	3	48	JER, MIX	FYN	<28 days							
EC7	113-3598-1	LVK	10	1	O101:H10	AslA **chuA**<br>cia csgA<br>eilA<br>espY2:000868321 fimH gad<br>hlyA hlyE<br>nlpI terC<br>traT yehA<br>yehB yehC<br>yehD	NG	2	6	HOL, MIX	JUT	7	+++	Massive	31		18		
EC8	114-3600-1	LVK	10	2	O101:H10	csgA espP<br>faeD faeI<br>fimH **fyuA**<br>gad hlyE hra<br>irp2 **iss** iucC<br>**iutA** lpfA<br>mcmA nlpI<br>**ompT**<br>**papA_F48**<br>**papC** sitA<br>terC traJ traT<br>yehA yehB<br>yehC yehD	NG	2	7	HOL, MIX	JUT	7	+	Massive	22		28		
EC9	115-5285-1	LVK	5911	1	O132:H18	AslA air<br>**chuA** eilA<br>espP faeI<br>fdeC fimH<br>**fyuA** gad<br>hlyE hra irp2	NG	24	60	JER, MIX	JUT	5	++	Massive	28				

						**iss** iucC **iutA** kpsE kpsMIII_K96 lpfA mcmA nlpI **ompT** **papA_F11** **papC** sitA sitA terC traJ traT yehA yehB yehC yehD													
EC10	176	LVK	58	4	O99a:H25	AslA **afaA** **afaB** **afaC** **afaD** air **chuA** eilA espP fdeC fimH **fyuA** gad hlyE iha irp2 **iss** iucC **iutA** kpsE kpsMIII_K98 lpfA mchB mchC mchF mcmA nlpI **ompT** shiA terC tia traT yehA yehB yehC yehD	ExPEC	22	54	HOL, MIX	FYN	7	+	Massive					
EC11	183	LVK	69	4	O15:H18	csgA fdeC fimH gad hlyE lpfA nlpI terC terC yehA yehB yehC yehD	ExPEC	22	54	HOL, MIX	FYN	7	+	Massive					
EC12	179	LVK	69	4	O15:H18	csgA fdeC fimH gad hlyE lpfA nlpI terC yehA yehB yehC yehD	DAEC/ APEC	22	54	HOL, MIX	FYN	7	+	Massive					
EC13	217	LVK	1485	2	O99a:H25	anr csgA fdeC fimH gad hha hlyE lpfA nlpI **ompT** terC traJ traT yehA yehB yehC yehD	NG	22	55	HOL, MIX	FYN	13	+++	Massive	24				
EC14	207	LVK	1725	1	O8:H21	AslA **afaA** **afaB** **afaC** **afaD** cea cia cib csgA espP faeD faeI faeJ fimH gad hha hlyE iha **iss** mchB mchC mchF nlpI terC traJ traT yehA yehB yehC yehD	NG	13	38	HOL, MIX	JUT	8	++	Massive					
EC15	218	LVK	351	1	O18ac:H17	anr csgA espP faeD faeI fimH gad iss	NG	13	39	HOL, MIX	JUT	3	+	Massive	23				

						nlpI terC traJ traT yehA yehB yehC yehD													
EC16	13109-1	LVK	108	4	O13/O129:H11	AslA afaA afaB afaC afaD air anr cea cia csgA espP faeD faeI fimH gad **iss** lpfA mchB mchC mchF nlpI terC traJ traT yehA yehB yehC yehD	DAEC	25	8	JER, MIX	JUT	12	++	Massive					
EC17	13109-4	LVK	398	4	O8:H20	anr csgA espP faeI fdeC fimH gad hlyE **iss** iucC **iutA** lpfA nlpI **papC** sitA terC traJ traT **yehA yehB yehC yehD**	NG	25	8	JER, MIX	JUT	12	++	Massive					
EC18	13109-5	LVK	410	4	O3:H9	anr csgA fdeC fimH **fyuA** gad hlyE irp2 **iss** lpfA nlpI **ompT** terC traJ traT yehA yehB yehC yehD	NG	25	8	JER, MIX	JUT	12	++	Massive					
EC19	167-1	LVK	1049	3	O160:H10	**afaA afaB afaC afaD** cif csgA **eae-b01a-beta** efa1 **ehxA** espA espB espF espJ espP fdeC fimH **fyuA** gad hlyE iha irp2 **iss** katP lpfA mchB mchF nleB nleC nlpI **ompT stx2a-O157-SF-258-98** terC tir toxB traT yehA yehB yehC yehD **stx2**	ExPEC	23	57	HOL, RDM, MIX	ZEALAND	7	+++	Massive					
EC20	169-1	LVK	164	3	O10:H42	AslA anr csgA fimH gad hlyE nlpI terC traJ yehA yehB	NG	23	58	HOL, RDM, MIX	ZEALAND	7	+++	Massive					
EC21	191	LVK	219	1	O26:H11	AslA **afaA afaB afaC afaD** clpK1 c olE2 csgA espP fimH **fyuA** gad	DAEC/ EHEC	23	59	HOL, RDM, MIX	ZEALAND	7	+	Massive					

						hha hlyE iha irp2 **iss** iucC **iutA** mchB mchC mchF mcmA nlpI sitA terC terC traT yehA yehB yehC yehD													
EC22	184	LVK	34	5	O62:H30	AslA **afaA** **afaB** **afaC** **afaD** clpK1 colE2 csgA espP fimH **fyuA** gad hha hlyE iha irp2 **iss** iucC **iutA** mchB mchC mchF mcmA nlpI sitA terC traT yehA yehB yehC yehD	NG	15	42	HOL, MIX	JUT	14	+++	Moderate					
EC23	17410-1	LVK	5236	5	O45:H19	AslA afaA afaB afaC afaD clpK1 colE2 csgA espP fimH fyuA gad hha hlyE iha irp2 iss iucC iutA mchB mchC mchF mcmA nlpI sitA terC traT yehA yehB yehC yehD	DAEC/ ExPEC	7	15	HOL	JUT	20	+++	Massive	22				
EC24	17410-4	LVK	410	5	O3:H9	AslA **afaA** **afaB** **afaC** **afaD** air anr astA cea **chuA** eilA espP faeC faeD faeI faeJ fdeC fimH gad hlyE hra iha **iss** iucC **iutA** kpsMIII_K98 lpfA nlpI ompT sitA terC traJ traT yehA yehB yehC yehD	DAEC/E xPEC	7	16	HOL	JUT	20	+++	Massive	22				
EC25	17420-1	LVK	108	1	O176:H30	AslA **afaA** **afaB** **afaC** **afaD** astA **chuA** fimH gad hlyE hra **iss** kpsE **kpsMII** mcmA nlpI **papA_F48** **papC** sitA terC yehA	DAEC/E xPEC	7	17	HOL	JUT	8	+++	Massive	22				

						yehB yehC yehD													
EC26	19629-8127-1	SEGES	108	4	O13/O135:H15	AslA air anr **chuA** cilA espP faeD faeI faeJ fimH gad hlyE **iss** kpsE kpsMIII_K98 lpfA nlpI **ompT** sitA terC traJ traT yehA yehB yehC yehD	NG	5	9	MIX	FYN	<28 days							
EC27	19629-8201-1	SEGES	3995	4	O9:H21	fimH gad hlyE **iss** lpfA nlpI ompT sitA terC traJ traT yehA yehB yehC yehD	ExPEC (APEC)	5	10	MIX	FYN	<28 days							
EC28	19903-8655-1	SEGES	69	3	O15	**afaA** **afaB** **afaC** **afaD** anr astA cif colE2 csgA **eae-b01a-beta** efa1 **ehxA** espA espB espF espJ espP fdeC fimH **fyuA** gad hlyE hra iha irp2 **iss** katP lpfA nleA nleB nleC nlpI **ompT** shiA **stx2a-O157-SF-258-98** tccP terC tir toxB traJ traT yehA yehB yehC yehD **stx2**	DAEC/ExPEC	4	11	JER, MIX	FYN	<28 days							
EC29	19903-8655-4	SEGES	6118	3	O132:H18	AslA **afaB** **afaC** **afaD** astA cea **chuA** fimH **fyuA** gad hlyE hra irp2 **iss** kpsE **kpsMII** mcmA nlpI **papA_F48** **papC** sitA terC yehA yehB yehC yehD	NG	4	11	JER, MIX	FYN	<28 days							
EC30	19903-8661-1	SEGES	69	1	O17/077:H18	anr csgA fimH gad hlyE lpfA nlpI **ompT** terC traJ traT yehA	NG	4	12	JER, MIX	FYN	<28 days							

						yehB yehC<br>yehD													
EC31	19903-<br>8700-1	SEGE<br>S	108	4	O40:H32	AslA aalH<br>cia csgA<br>**estap-STa1**<br>faeF faeI<br>**fanA fanB**<br>**fanC fanD**<br>**fanE fanF**<br>**fanG fanH**<br>**fimF41** fimH<br>gad hlyE hra<br>**iss** nlpI terC<br>traT yehA<br>yehB yehC<br>yehD	DAEC/E<br>HEC	4	13	JER,<br>MIX	FYN	<28<br>days							
EC32	19903-<br>8700-3	SEGE<br>S	219	4	O149	AslA aalH<br>cia csgA<br>**estap-STa1**<br>faeF faeI<br>**fanA fanB**<br>**fanC fanD**<br>**fanE fanF**<br>**fanG fanH**<br>**fimF41** fimH<br>gad hlyE hra<br>**iss** nlpI terC<br>traT yehA<br>yehB yehC<br>yehD	NG	4	13	JER,<br>MIX	FYN	<28<br>days							
EC33	19903-<br>8702-2	SEGE<br>S	69	1	O15	AslA **afaA**<br>**afaB afaC**<br>**afaD** astA<br>**chuA** csgA<br>fimH gad<br>hlyE hra **iss**<br>kpsE **kpsMII**<br>mcmA nlpI<br>**papC** shiA<br>sitA terC yehA<br>yehB yehC<br>yehD	DAEC/<br>ExPEC	4	14	JER,<br>MIX	FYN	<28<br>days							
EC34	19903-<br>8703-1	SEGE<br>S	174	1	O103:H2	AslA csgA<br>fimH gad hlyE<br>**iss** nlpI terC<br>yehA yehB<br>yehC yehD	DAEC/<br>EHEC	4	15	JER,<br>MIX	FYN	<28<br>days							
EC35	19903-<br>8703-2	SEGE<br>S	29	1	O26:H11	AslA **afaA**<br>**afaB afaC**<br>**afaD** anr<br>astA cea<br>**chuA** csgA<br>faeI fdeC<br>fimH **fyuA**<br>gad hlyE<br>ireA irp2 **iss**<br>iucC **iutA**<br>lpfA mcmA<br>nlpI **ompT**<br>**papA_F11**<br>**papC** pic<br>sitA terC **vat**<br>yehA yehB<br>yehC yehD	EHEC	4	15	JER,<br>MIX	FYN	<28<br>days							

EC36	19903-8705-1	SEGES	1049	1	O117:H10	AslA **afaA** **afaB** **afaC** **afaD** air anr **chuA** eilA espP fdeC fimH **fyuA** gad hlyE iha irp2 **iss** iucC **iutA** kpsMIII_K98 lpfA mchB mchC mchF mcmA nlpI **ompT** **papA_F19** sitA terC tia traJ traT yehA yehB yehC yehD	NG	4	16	JER, MIX	FYN	<28 days							
EC37	3844-2	LVK	69	1	O17/O44:H18	AslA **afaA** **afaB** **afaC** **afaD** cea clpK1 csgA espP fimH **fyuA** gad hha hlyE iha irp2 **iss** iucC **iutA** mchB mchC mchF mcmA nlpI **papA_F19** shiA sitA terC traJ traT yehA yehB yehC yehD	DAEC/ExPEC	6	14	HOL	JUT	9	+++	Massive	27		33		
EC38	45878-5518-1	SEGES	69	3	O15:H18	csgA espP faeD faeI fimH **fyuA** gad hlyE irp2 **iss** lpfA nlpI **ompT** **papC** terC traT	DAEC	10	23	JER, MIX	JUT	<28 days							
EC39	45878-5525-1	SEGES	69	4	O15:H18	AslA csgA espY2:000868321 fimH gad hlyE nlpI terC yehA yehB yehC yehD	DAEC	10	24	JER, MIX	JUT	<28 days							
EC40	45878-5526-1	SEGES	58	3	O86:H19	astA cba cma colE4 colE6 colE7 csgA fdeC fimH gad hlyE hra **iss** lpfA nlpI **ompT** terC traT yehA yehB yehC yehD	DAEC/ExPEC	10	25	JER, MIX	JUT	<28 days							
EC41	45878-5528-1	SEGES	58	3	O86:H19	anr csgA fdeC fimH gad hlyE lpfA nlpI	DAEC/ExPEC	10	26	JER, MIX	JUT	<28 days							

						**ompT** terC traJ traT yehA yehB yehC yehD													
EC42	47972-5908-2	SEGES	32	2	O145	AslA cdt-VB chuA csgA eilA espY2:000868321 fimH gad hlyE nlpI **ompT** shiB terC traJ traT yehA yehB yehC yehD	EHEC	1	29	HOL, MIX	JUT	<28 days							
EC43	47972-5909-1	SEGES	155	2	H51	AslA astA csgA fimH gad hlyE **iss** neuC nlpI terC yehA yehB yehC yehD	NG	1	30	HOL, MIX	JUT	<28 days							
EC44	47972-5910-1	SEGES	167	1	O101:H9	AslA csgA fimH gad hlyE iss nlpI terC yehA yehB	DAEC/ ExPEC (APEC)	1	31	HOL, MIX	JUT	<28 days							
EC45	47972-5911-2	SEGES	155	2	H21	csgA fdeC fimH gad hlyE **iss** lpfA nlpI **ompT** terC traJ traT yehA yehB yehC yehD	DAEC/APEC	1	32	HOL, MIX	JUT	<28 days							
EC46	47972-5912-1	SEGES	117	2	O33:H4	anr capU csgA espP faeC faeI faeJ fdeC **fyuA** gad hha hlyE irp2 **iss** lpfA nlpI **ompT** terC traJ traT yehA yehB yehC yehD	EHEC	1	33	HOL, MIX	JUT	<28 days							
EC47	47972-5913-1	SEGES	117	1	O33:H4	anr capU csgA espP faeC faeD faeI faeJ **fyuA** gad hha hlyE irp2 **iss** lpfA nlpI **ompT** terC traJ traT yehA yehB yehC yehD	DAEC/ ExPEC	1	34	HOL, MIX	JUT	<28 days							
EC48	47972-5914-2	SEGES	69	3	O15	anr capU csgA fimH gad hlyE nlpI terC traJ traT yehA yehB yehC yehD	DAEC/ExPEC	1	35	HOL, MIX	JUT	<28 days							
EC49	47972-5916-1	SEGES	38	4	O7	anr capU csgA fimH gad hlyE nlpI	NG	1	37	HOL, MIX	JUT	<28 days							

						terC traJ traT<br>yehA yehB<br>yehC yehD													
EC50	209	LVK	362	1	0154:H30	**F17A** **F17C**<br>**F17D** **F17G**<br>**afaA** **afaB**<br>csgA fdeC<br>fimH gad<br>hlyE **iss** lpfA<br>nlpI **ompT**<br>terC yehA<br>yehB yehC<br>yehD	DAEC/ExPEC	17	44	HOL, MIX	JUT	7	+	Moderate					
EC51	190	LVK	69	2	O17/O44:H18	**F17C** **F17D**<br>**F17G** capU<br>cdt-IIIB cnf2<br>csgA fimH<br>gad hlyE<br>iucC **iutA**<br>lpfA nlpI<br>**ompT** terC<br>traJ traT<br>yehA yehB<br>yehC yehD	NG	21	53	HOL, MIX	FYN	7	+	Massive	24				
EC52	92-1	LVK	58	3	O8:H10	csgA espP<br>faeC faeD faeI<br>fimH **fyuA**<br>gad hlyE hra<br>ireA irp2 **iss**<br>iucC **iutA**<br>lpfA mcmA<br>nlpI **ompT**<br>**papA_F48**<br>**papC** sitA<br>terC traJ traT<br>yehA yehB<br>yehC yehD	NG	26	12	HOL, MIX	JUT	10	++	Massive		25	31		29
EC53	9227-1	LVK	219	2	O26:H11	cofC cofD<br>cofE cofF<br>cofG cofH<br>cofI cofJ<br>cofP cofR<br>cofS cofT<br>csgA fdeC<br>fimH **fyuA**<br>gad hlyE irp2<br>**iss** lpfA nlpI<br>**ompT** terC<br>yehA yehB<br>yehC yehD	DAEC/EHEC	8	19	HOL	JUT	13	+++	Massive	23				
EC54	9229-5	LVK	362	2	O21:H9	**F17A** **F17C**<br>**F17D** **F17G**<br>anr csgA<br>fimH gad<br>hlyE **iss** nlpI<br>sitA terC traJ<br>traT yehA<br>yehB yehC<br>yehD	DAEC/ExPEC	8	20	HOL	JUT	10	+	Massive	26	35			
EC55	9232-1	LVK	187	1	O110:H2	AslA **afaA**<br>**afaB** **afaC**<br>**afaD** astA<br>**chuA** eilA<br>fimH gad	NG	8	21	HOL	JUT	2	+	Massive					

						hlyE hra **iss** iucC **iutA** kpsE **kpsMII_K5** nlpI **ompT** **papA_F11** **papC** shiA sitA terC tia yehA yehB yehC yehD													
EC56	9233-2	LVK	10	1	O101:H9	csgA fimH gad hlyE lpfA nlpI terC yehA yehB yehC yehD	ETEC	8	22	HOL	JUT	2	+++	Massive		21			
EC57	94-1	LVK	10	1	O101:H9	anr cif csgA **eae-e01-epsilon** efa1 **ehxA** espA espB espF espJ etpD fdeC fimH gad hlyE iha **iss** iucC **iutA** nleA nleB nleC nlpI **ompT** **stx1a-O157-FLY16** **stx2a-O157-SF-258-98** terC tir traT yehA yehB yehC yehD **stx1** **stx2**	ETEC	26	13	HOL, MIX	JUT	10	+++	Massive		20	19		
EC58	229	LVK	108	4	O107:H30	csgA fimH gad hlyE lpfA nlpI terC yehA yehB yehC yehD	DAEC/ExPEC	19	48	HOL, MIX	JUT	12	+++	Massive	27				
EC59	213	LVK	108	4	O107:H54	csgA fimH gad hlyE **iss** lpfA nlpI **ompT** terC yehA yehB yehC yehD	NG	19	48	HOL, MIX	JUT	12	+++	Massive	27				
EC60	215	LVK	602	4	O15:H4	AslA, anr, csgA, fdeC, fimH, hha, hlyE, nlpl, terC, traJ, traT, yehA, yehB, yehC, yehD	DAEC/ ExPEC (UPEC)	19	48	HOL, MIX	JUT	12	+++	Massive	27				
EC61	214	LVK	69	1	O15:H18	AslA, **afaA, afaB, afaC, afaD**, csgA, fdeC, fimH, gad, hlyE, hra, ireA, **iroN**, iss, iucC, **iutA**, mcbA, mchB, mchC, mcmA, nlpl,	DAEC/ExPEC	19	49	HOL, MIX	JUT	10	+	Massive	29				

						**papA_F48**, **papC**, shiA, sitA, terC, tia, yehA, yehB, yehC, yehD													
EC62	232	LVK	108	1	O107:H30	AslA, cea, csgA, fdeC, fimH, gad, hha, hylE, iss, iucC, **iutA**, nlpl, **ompT**, sitA, terC, traJ, traT, yehA, yehB, yehC, yehD	DAEC/ ExPEC	19	49	HOL, MIX	JUT	10	+	Massive	29				
EC63	182	LVK	23	4	O32:H9	AslA, aalH, cia, csgA, etpD, faeC, faeD, faeF, faeI, **fanA**, fanB, fanE, fanH, fdeC, **fim41**, fimH, gad, hha, hlyE, hra, **iss**, nlpl, terC, traT, yehA, yehB, yehC, yehD	NG	19	50	HOL, MIX	JUT	3	+++	Moderate	22				
EC64	196	LVK	4701	5	O109:H45	AslA, aalH, cia, csgA, etpD, faeC, faeD, faeF, faeH, faeI, **fanA**, fanB, fanC, fanE, fanH, fdeC, **fimF41**, fimH, gad, hha, hlyE, hra, **iss**, nlpl, terC, traT, yehA, yehB, yehC, yehD	NG	18	45	HOL, MIX	JUT	4-11 days	+	Weak	27				
EC65	231	LVK	446	5	O88:H8	astA, cea, cif, cnf3, **eae-b01a-beta**, efa1, **ehxA**, espA, espB, espF, espI, espJ, fdeC, fimH, **fyuA**, gad, hha, hylE, iha, irp2, **iss**, iucC, **iutA**, **lpfA**, nleA, nleB, nleC, nlpl, **ompT** tccP, terC, tir, traT, tsh, yehA, yehB, yehC, yehD	NG	18	45	HOL, MIX	JUT	4-11 days	+	Weak	27				
EC66	203	LVK	5756	5	O117:H16	**stx2**, **astA**, cba, cif, cma, csgA, **eae-b10-beta**, efa1, **ehxA**, espA, espB, espF, espJ, etpD, fdeC, fimH, **fyuA**,	NG	18	45	HOL, MIX	JUT	4-11 days	+	Weak	27				

						gad, hha, hlyE, iha, irp2, **iss**, iucC, **iutA**, lpfA, nleA, nleB, nlpl, **ompT**, **stx2a-O157-EDL933**, **stx2a-ONT-EK9900**, terC, tir, traJ, traT, yehA, yehB, yehC, yehD													
EC67	194	LVK	3381	5	O50:H25	AslA,csgA, fdeC, fimH, **fyuA**, hlyE, irp2, nlpl, shiA, terC, yehA, yehB, yehC	NG	18	45	HOL, MIX	JUT	4-11 days	+	Weak	27				
EC68	211	LVK	145	4	O108	AslA, csgA, fdeC, fimH, **fyuA**, hlyE, irp2, nlpl, shiA, terC, yehA, yehB, yehC	NG	18	46	HOL, MIX	JUT	4	++	Weak					
EC69	233	LVK	1695	4	O52:H10	**F17A, F17C, F17D, F17D,** astA, csgA, fdeC, fimH, gad, hha, hlyE, hra, irp2, **iss**, lpfA, nlpl, **ompT**, shiA, terC, yehA, yehB, yehC, yehD	NG	18	46	HOL, MIX	JUT	4	++	Weak					
EC70	178	LVK	1304	4	O91:H7	**afaA, afaB, afaC, afaD**, anr, csgA, espP, fdeC, fimH, gad, hha, hylE, iha, **iss**, iucC, **iutA**, kpsE, kpsMIII_K98, lpfA, mchB, mchC, nlpl, shiA, terC, tia, traJ, traT, yehA, yehB, yehC, yehD	NG	18	46	HOL, MIX	JUT	4	++	Weak					
EC71	216	LVK	58	5	H27	anr, astA, cea, cia, csgA, evaC, etsC, fdeC, fimH, **fyuA**, hylE, **hlyF**, hra, ireA, **iroN**, irp2, **iss**, iucC, **iutA**, lpfA, mchF, mcmA, nlpl, **ompT**, **papA_F48, papC**, sitA, terC, traJ, traT,	NG	18	47	HOL, MIX	JUT	6	+	Weak					29

						yehA, yehB, yehC, yehD													
EC72	221	LVK	58	5	H27	anr, cia, cia, csgA, evaC, etsC, fdeC, fimH, **fyuA**, gad, hha, hlyE, **hlyF**, **iroN**, irp2, **iss**, iucC, **iutA**, lpfA, mchF, nlpl, **omp**T, sitA, terC, traJ, traT, yehA, yehB, yehC, yehD	NG	18	47	HOL, MIX	JUT	6	+	Weak					29
EC73	208	LVK	50	5	O150:H8	**F17A, F17C, F17D, F17G**, csgA, fdeC, fimH, gad, hlyE, **iss**, lpfA, nlpl, **ompT**, shiB, terC, traT, yehA, yehB, yehC, yehD	NG	18	47	HOL, MIX	JUT	6	+	Weak					29
EC74	204	LVK	38	5	O7	anr, colE7, csgA, fdeC, fimH, hha, hlyE, lpfA, mrkA:ABW83988, nlpl, terC, traJ, traT, yehA, yehB, yehC, yehD	NG	18	47	HOL, MIX	JUT	6	+	Weak					29
EC75	177	LVK	2522	1	O25:H8	AslA, aalF,**afaA, afaB, afaC, afaS**, air, anr, **chuA**, csgA, eilA, espP, faeC, faeD, faeF, faeI, faeJ, fdeC, fimH, **fyuA**, gad, hha, hlyE, irps2, **iss**, iss, **kpsE, kpsMII_K96**, ipfA, MCHf,nlpl, **ompT**, shiB, sitA, terC, traJ, traT, yehA, yehB, yehC, yehD	(DAEC)	14	40	HOL, MIX	JUT	19	+	Massive					
EC76	225	LVK	641	1	O121:H10	AslA, aalF, **afaA, afaB, afaC, afaD**, air, **chuA**, colE7, csgA, eilA, espP, faeC, faeD, faeF, faeI, faeJ, fdeC, fimH, **fyuA**, gad, hha, hlyE, iha, irp2, **iss**,	NG	14	41	HOL, MIX	JUT	14	+	Massive					32

						**kpsE, kpsMII_K96,** lpfA, mchB, mchC, mchF, mcmA, nlpl, **ompT, papA_F48, papC**, shiA, sitA, terC, tia, traJ, trsT, yehA, yehB, yehC, yehD													
EC77	19903-8653-2	SEGES	69	3	H15	aalF, **afaA, afaB, afaC, afaD**, anr, astA, cea, cia, csgA, espP, faeC, faeD, faeF, faeI, fdeC, fimH, **fyuA**, gad, hylE, hra, irp2, iucC, **iutA**, lpfA, mdhB,mchc, mchF, nlpl, **ompT, papA_F48,papC**, sitA, terC, traJ, traT, yehA, yehB, yehC, yehD	DAEC/ ExPEC	4	11	JER, MIX	FYN	<28 days							
EC78	206	LVK	58	2	O9:H25	**afaA, afaB, afaC, afaD**, cia, csgA, fdeC, fimH, **fyuA**, gad, hha, hylE, hra, ireA, irp2, **iss**, iucC, **iutA**, lpfA, mcmA, nlpl, **ompT, papA_F48, papC**, shiA, sitA, terC, tia, traT, yehA, yehB, yehC, yehD	ExPEC	22	56	HOL, MIX	FYN	5	++	Massive					
EC79	14200-1	LVK	167	3	O101:H9	csgA, fdeC, fimH, gad, hlyE, **iss**, **lpfA**, nlpl, **ompT, papC**, sitA , terC, yehA, yehB, yehC, yehD	NG	11	28	HOL	JUT	7	++	massive			32		
EC80	45878-5520-4	SEGES	5880		H7	csgA, fdeC, fimH, gad, hlyE, **iss**, **lpfA**, nlpl, **ompT, papC**, sitA, terC, yehA, yehB, yehC, yehD	NG	10		JER, MIX	JUT	<28 days							
EC81	14200-4	LVK	2325	3	O154:H25	**afaA, afaB, afaC, afaD**,	NG	11	28	HOL	JUT	7	++	massive			32		

						anr, astA, cia, csgA, evaC, etsC, fdeC, fimH, gad, hylE, **hlyF, iroN, iss,** iucC, **iutA,** lpfA, mchF, neuC, nlpl, **ompT**, shiA, sitA, terC, tia, traJ, traT, yehA, yehB, yehC, yehD													
EC82	189	LVK	362	1	O153	aalF, anr, astA, cea, csgA, espP, faeC, faeD, faeF, faeI, faeJ, fdeC, fimH, **fyuA**, hlyE, hra, irp2, iucC, **iutA,** lpfA, mcmA, nlpl, **ompT, papA_F48, papC,** sitA, terC, traJ, traT, yehA, yehB, yehC, yehD	DAEC/ ExPEC	20	52	HOL, RDM, MIX	JUT	8	++	Massive	26		24		
EC83	45878-5521-5	SEGE S	88	3	O8:H4	**afaA, afaB, afaC, afaD**, anr, cia, csgA, fdeC ,fimH, fyuA, gad, hha, hlyE, hra, ireA, irp2, **iss**, iucC, **iutA,** lpfA, mcmA, nlpl, **ompT, papA_F48, papC**, shiA, terC, tia, traT, yehA, yehB, yehC, yehD	NG	10	28	JER, MIX	JUT	<28 days							
EC84	227	LVK	155	2	H21	aalF, **afaA, afaB, afaC, afaD**, anr, astA, cia, csgA, espP, faeC, faeD, faeF, faeI, fdeC, fimH, gad, hha, hlyE, hra, iucC, **iutA,** lpfA, mcmA, nlpl, **ompT, papA_F48, papC**, shiA, sitA, terC, tia, traJ, traT, yehA, yehB, yehC, yehD	NG	19	51	HOL, MIX	JUT	7	+++	Massive					
EC85	205	LVK	17	1	O103:H2	aalF, **afaA, afaB, afC, afaD**, anr,	EHEC	16	43	HOL, MIX	JUT	14	+	Massive	33		32		34

						**astA**, capU, csgA, espP, faeC, faeD, faeF, faeI, faeJ, fdeC, fimH, gad, hha, hlyE, hra, iha, iucC, **iutA,** lpfA, mcmA, nlpl, **ompT, papA_F48, papC**, sitA, terC, traJ, traT, yehA, yehB, yehC, yehD													
EC86	45878-5524-3	SEGES	10	4	O101:H9	**F17A, F17C, F17D, F17G**, **afaA, afaB**, cib, csgA, fdeC, fimH, **fyuA**, hha, hlyE, hra, irp2, **iss,** lplfA, nlpl, **ompT**, shiB, terC, traT, yehA, yehB, yehC, yehD	NG	10	29	JER, MIX	JUT	<28 days							
EC87	45878-5524-4	SEGES	398	4	O133:H20	AslA, **afaA, afaB, afaC, afaD**, astA, **chuA**, csgA, fdeC, fimH, gad, hha, hlyE, hra, **iss,** kpsE, **kpsMII, kpsMII_K5**, mcmA, nlpl, **papA_F48, papC**, sitA, terC, yehA, yehB, yehC, yehD	NG	10	29	JER, MIX	JUT	<28 days							
EC88	45878-5522-1	SEGES	21	1	O26:H4	AslA, **afaA, afaB, afaC, afaD**, astA, **chuA**, csgA, fdeC, fimH, gad, hha, hlyE, hra, kpsE, **kpsMII, kpsMII_K5**, mcmA, nlpl, **papA_F48, papC**, sitA, terC, yehA, yehB, yehC, yehD	EHEC	10	30	JER, MIX	JUT	<28 days							
EC89	45878-5520-4	SEGES	109	4	O28:H8	AslA, **afaA, afaB, afaC, afaD**, **astA**, cea, **chuA**, csgA, fdeC, fimH, **fyuA**, gad, hha, hlyE,	NG	10	27	JER, MIX	JUT	<28 days							

						hra, irp2, **iss**, kpsE, **kpsMII**, mcmA, nlpl, **papA_F48, papC**, sitA, terC, yehA, yehB, yehC, yehD													
EC90	131-5938-1	LVK	5177	2	O88:H25	anr, astA, cia, csgA, evaC, etsC, fdeC, fimH, hha, hlyE, **hlyF, iroN, iss**, iucC, **iutA**, mchF, nlpl, **ompT, papC**, shiA, sitA, terC, tia, traJ, traT, yehA, yehB, yehC, yehD	NG	8	18	HOL	JUT	2	++	Massive					
EC91	131-5938-2	LVK	939	2	H23	aalF, **afaA, afaB, afaC, afaD**, csgA, espP, faeC, faeD, faeF, faeI,fdeC, fimH, **fyuA**, gad, hha, hylE, iha, irp2, **iss**, iucC, nlpl, sitA, terC, traJ, traT, yehA, yehB, yehD	NG	8	18	HOL	JUT	2	++	Massive					
EC92	45878-5520-1	SEGES	5880	4	O75:H7	aalF, **afaA, afaB, afaC, afaD**, anr, csgA, espP, faeC, faeFD, faeF, faeI, faeJ, fdeC, fimH, **fyuA**, gad, hha, hylE, iha, irp2, **iss**, iucC, **iutA**, nlpl, sitA, terC, traJ, traT, yehA, yehB, yehD	NG	10	27	JER, MIX	JUT	<28 days							
EC93	45878-5520-2	SEGES	17734	4	O26	aalF, anr, csgA, espA, faeC, faeD, faeF, faeI, fdeC, fimH, gad, hylE, **iss**, lpfA, nlpl, **ompT, papC**, terC, traJ, traT, yehA, yehB, yehC, yehD	NG	10	27	JER, MIX	JUT	<28 days							
EC94	K2368	VetLab	10		O101:H10	AslA, air, **chuA**, csgA, eilA, espY2, fdeC, fimH, gad, hylE, **iss**, kpsE, **kpsMII_K5**,	ETEC	27	72	HOL	JUT								

						lpfA, nlpl, **papC**, sitA, terC, yehA, yehB, yehC, yehD, **yfcV**													
EC95	K2633	VetLab	10		O101:H9	aalF, anr, cia, csgA, espP, faeC, faeD, faeF, faeI, faeJ, fdeC, fimH, gad, hha, hlyE, **iss**, lpfA, nlpl, **papC**, sitA, terC, traJ, traT, yehA, yehB, yehC, yehD	ETEC	28	73	HOL	JUT								
EC96	23-33	VetLab	10		O101:H9	AslA, cib, csgA, fdeC, fimH, gad, hlyE, nlpl, terC, traT, yehA, yehB, yehC, yehD	ETEC-tox-	28	74	HOL	JUT								
EC97	10075	VetLab	10		O101:H9	anr, csgA, fimA, gad, hlyE, **iss**, nlpl, sitA, terC, traT, traJ, yehA, yehB, yehC, yehD	ETEC	29	75	HOL	JUT								
EC98	9988	VetLab	10		O101:H9	astA,csgA, fdeC, fimH, hha, hylE, iss, nlpl,sit, terC, yehD	ETEC	29	76	HOL	JUT								
EC99	1123	VetLab	10		O101:H9	AslA, aalC, **afaA, afaB**, air, cea, colE2, csgA, eilA, etsC, faeC, faeF, faeI, faeJ, fdeC, fimH, gad, hlyF, iha, iss, iucC, **iutA**, kpsE, **kpsMII_K5**, lpfA, mchB, mchF, mcmA, nlpl, **ompT, papA_F48, papC**, shiA, sitA, terC, tia, traJ, traT, yehA, yehB, yehC, yehD, **yfcV**	ETEC	29	77	HOL	JUT								
EC100	K2859	VetLab	21		O26:H11	AslA **chuA** espY2:000868321 fdeC fimH gad hlyE **iss** kpsE **kpsMII** lpfA nlpI **ompT** terC terC terC	EHEC	29	78	HOL	JUT								

						yehB yehC<br>yehD **yfcV**													
EC101	21-5	GUDP	21		O26:H11	AslA anr capU<br>csgA gad hyE<br>iha **iss** mcmA<br>nlpI **papA_F19**<br>**papA_F48**<br>**papC** shiA<br>terC terC tia<br>traJ traT traT<br>yehA yehB<br>yehC yehD	EHEC	29	79	HOL	JUT								
EC102	K116980	VetLab	58		O45:H2	**afaA** **afaB**<br>**afaC** **afaD**<br>anr astA<br>capU csgA<br>espP faeI<br>fimH gad<br>hlyE hra iha<br>iucC **iutA**<br>lpfA mcmA<br>nlpI **ompT**<br>**papA_F19**<br>**papA_F48**<br>**papC** shiA<br>sitA terC<br>tia traJ traT<br>yehA yehB<br>yehC yehD	ExPEC<br>(APEC)	29	80	HOL	JUT								
EC103	K117637	VetLab	58		O9	AslA air anr<br>**chuA**<br>cvaC eilA<br>etsC fdeC<br>fimH **fyuA**<br>gad hlyE **hlyF**<br>hra **iroN** irp2<br>**iss** iucC **iutA**<br>kpsE<br>**kpsM_K15**<br>lpfA mchF<br>nlpI **ompT**<br>**papA_F48**<br>**papC** sitA<br>terC traT<br>tsh yehA<br>yehB yehC<br>yehD	NG	30	84	HOL,<br>JER,<br>MIX	JUT								
EC104	9-1	GUDP	58		O8:H10	AslA anr<br>capU csgA<br>gad hlyE<br>hra iha **iss**<br>mcbA cbA<br>nlpI<br>**papA_F19**<br>**papA_F48**<br>**papC** terC<br>tia traJ<br>traT yehA<br>yehB yehC<br>yehD	ExPEC<br>(APEC)	30	81	HOL,<br>JER,<br>MIX	JUT								
EC105	18-1	GUDP	58		O25:H28	AslA anr<br>capU csgA<br>gad hlyE hra<br>iha **iss** mcmA	DAEC/<br>ExPEC	30	82	HOL,<br>JER,<br>MIX	JUT								

						nlpI **papA_F19** **papA_F48** **papC** terC tia traJ traT yehA yehB yehC yehD													
EC106	18-3	GUDP	58		O25:H28	AslA **chuA** cia csgA eilA espY2:000868321 fimH gad hlyA hlyE nlpI terC traT yehA yehB yehC yehD	DAEC/ ExPEC	30	83	HOL, JER, MIX	JUT								
EC107	K117871	VetLab	88		O8	csgA espP faeD faeI fimH **fyuA** gad hlyE hra irp2 **iss** iucC **iutA** lpfA mcmA nlpI **ompT** **papA_F48** **papC** sitA terC traJ traT yehA yehB yehC yehD	DAEC	30	85	HOL, JER, MIX	JUT								
EC108	K1732	VetLab	88		O8:H9	AslA air **chuA** eilA espP faeI fdeC fimH **fyuA** gad hlyE hra irp2 **iss** iucC **iutA** kpsE kpsMIII_K96 lpfA mcmA nlpI **ompT** **papA_F11** **papC** sitA sitA terC traJ traT yehA yehB yehC yehD	NG	7	61	HOL	JUT								
EC109	K7051-2	VetLab	88		O9a:H17	AslA **afaA** **afaB** **afaC** **afaD** air **chuA** eilA espP fdeC fimH **fyuA** gad hlyE iha irp2 **iss** iucC **iutA** kpsE kpsMIII_K98 lpfA mchB mchC mchF mcmA nlpI **ompT** shiA terC tia traT yehA yehB	ExPEC (APEC)	7	62	HOL	JUT								

						yehC yehD													
EC110	23-5	VetLab	88		O8:H17	csgA fdeC fimH gad hlyE lpfA nlpI terC terC yehA yehB yehC yehD	DAEC/ ExPEC	7	63	HOL	JUT								
EC111	K116726	VetLab	117		O119:H4	csgA fdeC fimH gad hlyE lpfA nlpI terC yehA yehB yehC yehD	DAEC/ ExPEC (UPEC)	7	64	HOL	JUT								
EC112	K117022	VetLab	117		H4	anr csgA fdeC fimH gad hha hlyE lpfA nlpI **ompT** terC traJ traT yehA yehB yehC yehD	DAEC/ ExPEC	7	65	HOL	JUT								
EC113	k116853	VetLab	117		H6	AslA **afaA afaB afaC afaD** cea cia cib csgA espP faeD faeI faeJ fimH gad hha hlyE iha **iss** mchB mchC mchF nlpI terC traJ traT yehA yehB yehC yehD	ExPEC (APEC)	32	86	HOL	JUT								
EC114	K4788-2	VetLab	117		O119:H4	anr csgA espP faeD faeI fimH gad iss nlpI terC traJ traT yehA yehB yehC yehD	ExPEC	32	87	HOL	JUT								
EC115	K117551	VetLab	120		-	AslA afaA afaB afaC afaD air anr cea cia csgA espP faeD faeI fimH gad **iss** lpfA mchB mchC mchF nlpI terC traJ traT yehA yehB yehC yehD	NG	32	88	HOL	JUT								
EC116	11020	VetLab	227		O9:H10	anr csgA espP faeI fdeC fimH gad hlyE **iss** iucC **iutA** lpfA nlpI **papC** sitA terC traJ traT **yehA yehB yehC yehD**	NG	32	89	HOL	JUT								
EC117	1183	VetLab	301		O80:H2	anr csgA fdeC fimH **fyuA**	EHEC/ DAEC	32	90	HOL	JUT								

						gad hlyE irp2 **iss** lpfA nlpI **ompT** terC traJ traT yehA yehB yehC yehD	/ExPEC (APEC)												
EC118	21-2	GUDP	611		O96:H4	**afaA afaB afaC afaD** cif csgA **eae-b01a-beta** efa1 **ehxA** espA espB espF espJ espP fdeC fimH **fyuA** gad hlyE iha irp2 **iss** katP lpfA mchB mchF nleB nleC nlpI **ompT stx2a-O157-SF-258-98** terC tir toxB traT yehA yehB yehC yehD **stx2**	NG	34	91	HOL	JUT								
EC119	21-3	GUDP	744		O101:H9	AslA anr csgA fimH gad hlyE nlpI terC traJ yehA yehB	NG	34	92	HOL	JUT								
EC120	K95572	VetLab	3042		O45:H25	AslA **afaA afaB afaC afaD** clpK1 c olE2 csgA espP fimH **fyuA** gad hha hlyE iha irp2 **iss** iucC **iutA** mchB mchC mchF mcmA nlpI sitA terC terC traT yehA yehB yehC yehD	NG	34	93	HOL	JUT								
EC121	K116952	VetLab	-		-	AslA **afaA afaB afaC afaD** clpK1 colE2 csgA espP fimH **fyuA** gad hha hlyE iha irp2 **iss** iucC **iutA** mchB mchC mchF mcmA nlpI sitA terC traT yehA yehB yehC yehD	NG	34	94	HOL	JUT								
EC122	180	LVK	10		O101:H9	AslA **afaA afaB**	NG	35	95	HOL	JUT	22	++	Massive					

EC123	193	LVK	10		O101/O9/ 9a:H9	AslA **afaA** **afaB** **afaC** **afaD** air anr astA cea **chuA** eilA espP faeC faeD faeI faeJ fdeC fimH gad hlyE hra iha **iss** iucC **iutA** kpsMIII_K98 lpfA nlpI ompT sitA terC traJ traT yehA yehB yehC yehD	DAEC/ ExPEC	36	96	HOL	JUT	15	+++	Massive	26				
EC124	220	LVK	10		O101:H9	AslA **afaA** **afaB** **afaC** **afaD** astA **chuA** fimH gad hlyE hra **iss** kpsE **kpsMII** mcmA nlpI **papA_F48** **papC** sitA terC yehA yehB yehC yehD	NG	37	97	JER	ZEALAND	6	++	Massive	33		22		
EC125	210	LVK	10		O101:H9	AslA air anr **chuA** eilA espP faeD faeI faeJ fimH gad hlyE **iss** kpsE kpsMIII_K98 lpfA nlpI **ompT** sitA terC traJ traT yehA yehB yehC yehD	F5 -tox	37	98	JER	ZEALAND	12	++	Massive	26				31
EC126	224	LVK	10		O101:H9	fimH gad hlyE **iss** lpfA nlpI ompT sitA terC traJ traT yehA yehB yehC yehD	F5 -tox	37	99	JER	ZEALAND	12	++	Massive	20				27
EC127	201	LVK	29		O26:H11	**afaA** **afaB** **afaC** **afaD** anr astA cif colE2 csgA **eae-b01a-beta** efa1 **ehxA** espA espB espF espJ espP fdeC fimH **fyuA** gad hlyE hra iha irp2 **iss** katP lpfA nleA nleB nleC nlpI	EPEC	38	100	JER	JUT	13	+++	Massive	27				

						**ompT** shiA **stx2a-O157-SF-258-98** tccP terC tir toxB traJ traT yehA yehB yehC yehD **stx2**													
EC128	183	LVK	29		O26:H11	AslA **afaB** **afaC** **afaD** astA cea **chuA** fimH **fyuA** gad hlyE hra irp2 **iss** kpsE **kpsMII** mcmA nlpI **papA_F48** **papC** sitA terC yehA yehB yehC yehD	EHEC	39	101	HOL	JUT	7	+++	Weak	30		18		
EC129	184	LVK	34		O99:H33	anr csgA fimH gad hlyE lpfA nlpI **ompT** terC traJ traT yehA yehB yehC yehD	NG	40	102	HOL	JUT	6	+	Massive					
EC130	189	LVK	34		O99:H33	AslA aalH cia csgA **estap-STa1** faeF faeI **fanA** **fanB** **fanC** **fanD** **fanE** **fanF** **fanG** **fanH** **fimF41** fimH gad hlyE hra **iss** nlpI terC traT yehA yehB yehC yehD	NG	13	68	HOL, MIX	JUT	7	+	Massive	24				
EC131	190	LVK	56		O23:H31	AslA aalH cia csgA **estap-STa1** faeF faeI **fanA** **fanB** **fanC** **fanD** **fanE** **fanF** **fanG** **fanH** **fimF41** fimH gad hlyE hra **iss** nlpI terC traT yehA yehB yehC yehD	NG	13	69	HOL, MIX	JUT	7	+	Massive	29				
EC132	191	LVK	58		O25:H28	AslA **afaA** **afaB** **afaC** **afaD** astA **chuA** csgA fimH gad hlyE hra **iss** kpsE **kpsMII**	DAEC/ ExPEC	13	70	HOL, MIX	JUT	7	+	Massive	24				

						mcmA nlpI<br>**papC** shiA<br>sitA terC yehA<br>yehB yehC<br>yehD													
EC133	193	LVK	58		O8:H25	AslA csgA<br>fimH gad hlyE<br>**iss** nlpI terC<br>yehA yehB<br>yehC yehD	ExPEC (APEC)	41	103	HOL	JUT	7	++	Massive	30		28		
EC134	194	LVK	58		O8:H25	AslA **afaA**<br>**afaB** **afaC**<br>**afaD** anr<br>astA cea<br>**chuA** csgA<br>faeI fdeC<br>fimH **fyuA**<br>gad hlyE<br>ireA irp2 **iss**<br>iucC **iutA**<br>lpfA mcmA<br>nlpI **ompT**<br>**papA_F11**<br>**papC** pic<br>sitA terC **vat**<br>yehA yehB<br>yehC yehD	ExPEC (APEC)	42	104	HOL	JUT	6	+	Massive	20		25		
EC135	195	LVK	58		O35:H21	AslA **afaA**<br>**afaB** **afaC**<br>**afaD** air anr<br>**chuA** eilA<br>espP fdeC<br>fimH **fyuA**<br>gad hlyE iha<br>irp2 **iss** iucC<br>**iutA**<br>kpsMIII_K98<br>lpfA mchB<br>mchC mchF<br>mcmA nlpI<br>**ompT**<br>**papA_F19**<br>sitA terC tia<br>traJ traT<br>yehA yehB<br>yehC yehD	NG	43	105	JER	FYN	7	+	Massive	27				
EC136	196	LVK	58		H37	AslA **afaA**<br>**afaB** **afaC**<br>**afaD** cea<br>clpK1 csgA<br>espP fimH<br>**fyuA** gad<br>hha hlyE<br>iha irp2 **iss**<br>iucC **iutA**<br>mchB mchC<br>mchF mcmA<br>nlpI<br>**papA_F19**<br>shiA sitA<br>terC traJ traT<br>yehA yehB<br>yehC yehD	NG	44		HOL	JUT	9	+	Massive	23				

EC137	197	LVK	69		O15:H6	csgA espP faeD faeI fimH **fyuA** gad hlyE irp2 **iss** lpfA nlpI **ompT** **papC** terC traT	DAEC/ ExPEC	44	108	HOL	JUT	10	+	Massive	23				
EC138	201	LVK	69		O15:H18	AslA csgA espY2:000868321 fimH gad hlyE nlpI terC yehA yehB yehC yehD	DAEC/ ExPEC	46	112	HOL	JUT	12	+	Weak	30		24		
EC139	203	LVK	88		O86:H25	astA cba cma colE4 colE6 colE7 csgA fdeC fimH gad hlyE hra **iss** lpfA nlpI **ompT** terC traT yehA yehB yehC yehD	DAEC/ ExPEC	45	109	HOL,MIX	JUT	7	+++	Weak					
EC140	204	LVK	88		O8:H17	anr csgA fdeC fimH gad hlyE lpfA nlpI **ompT** terC traJ traT yehA yehB yehC yehD	DAEC/ ExPEC	45	110	HOL,MIX	JUT	7	+	Moderate					
EC141	205	LVK	88		O8:H9	AslA cdt-VB chuA csgA eilA espY2:000868321 fimH gad hlyE nlpI **ompT** shiB terC traJ traT yehA yehB yehC yehD	NG	45	111	HOL, MIX	JUT	2	++	Moderate					
EC142	206	LVK	88		O8:H9	AslA astA csgA fimH gad hlyE **iss** neuC nlpI terC yehA yehB yehC yehD	NG	48	114	HOL, MIX	JUT	16	+++	Massive					
EC143	207	LVK	88		O8/O104:H10	AslA csgA fimH gad hlyE iss nlpI terC yehA yehB	DAEC/ ExPEC (APEC)	47	113	HOL	JUT	5	+++	Massive			31		
EC144	208	LVK	88		O86:H25	csgA fdeC fimH gad hlyE **iss** lpfA nlpI **ompT** terC traJ traT yehA yehB yehC yehD	ExPEC	49	117	HOL	JUT	5	+++	Massive					
EC145	209	LVK	88		O8:H17	anr capU csgA espP faeC faeI faeJ	DAEC/ ExPEC	49	118	HOL	JUT	16	+++	Massive					

						fdeC **fyuA**<br>gad hha<br>hlyE irp2 **iss**<br>lpfA nlpI<br>**ompT** terC<br>traJ traT<br>yehA yehB<br>yehC yehD													
EC146	210	LVK	88		O6/O32:H25	anr capU<br>csgA espP<br>faeC faeD<br>faeI faeJ<br>**fyuA** gad hha<br>hlyE irp2 **iss**<br>lpfA nlpI<br>**ompT** terC<br>traJ traT yehA<br>yehB yehC<br>yehD	DAEC/<br>ExPEC	48	115	HOL,<br>MIX	JUT	12	+++	Moderate					
EC147	211	LVK	88		O8/O32:H25	anr capU<br>csgA fimH<br>gad hlyE nlpI<br>terC traJ traT<br>yehA yehB<br>yehC yehD	DAEC/<br>ExPEC	48	116	HOL,<br>MIX	JUT	8	+	-					
EC148	213	LVK	223		O7:H4	anr capU<br>csgA fimH<br>gad hlyE nlpI<br>terC traJ traT<br>yehA yehB<br>yehC yehD	DAEC	50	119	HOL,<br>MIX	JUT	7	+	Massive	23				
EC149	214	LVK	362		O154:H30	**F17A F17C**<br>**F17D F17G**<br>**afaA afaB**<br>csgA fdeC<br>fimH gad<br>hlyE **iss** lpfA<br>nlpI **ompT**<br>terC yehA<br>yehB yehC<br>yehD	DAEC/<br>ExPEC	50	120	HOL,<br>MIX	JUT	12	+	Massive	30				
EC150	215	LVK	362		O154:H30	**F17C F17D**<br>**F17G** capU<br>cdt-IIIB cnf2<br>csgA fimH<br>gad hlyE<br>iucC **iutA**<br>lpfA nlpI<br>**ompT** terC<br>traJ traT<br>yehA yehB<br>yehC yehD	DAEC/<br>ExPEC	50	121	HOL,<br>MIX	JUT	7	+	Moderate	37				36
EC151	216	LVK	362		O21:H9	csgA espP<br>faeC faeD faeI<br>fimH **fyuA**<br>gad hlyE hra<br>ireA irp2 **iss**<br>iucC **iutA**<br>lpfA mcmA<br>nlpI **ompT**<br>**papA_F48**<br>**papC** sitA<br>terC traJ traT	DAEC/<br>ExPEC	50	122	HOL,<br>MIX	JUT	12	++	Weak					33

						yehA yehB yehC yehD													
EC152	217	LVK	540		O9/O9a:H30	cofC cofD cofE cofF cofG cofH cofI cofJ cofP cofR cofS cofT csgA fdeC fimH **fyuA** gad hlyE irp2 **iss** lpfA nlpI **ompT** terC yehA yehB yehC yehD	ExPEC (APEC)	50	123	HOL, MIX	JUT	9	++	Weak				37	
EC153	218	LVK	540		O9/9a:H9	**F17A F17C F17D F17G** anr csgA fimH gad hlyE **iss** nlpI sitA terC traJ traT yehA yehB yehC yehD	DAEC	31	126	JER	JUT	2	++	Moderate		20			
EC154	220	LVK	540		O9/9a:H9	AslA **afaA afaB afaC afaD** astA **chuA** eilA fimH gad hlyE hra **iss** iucC **iutA** kpsE **kpsMII_K5** nlpI **ompT papA_F11 papC** shiA sitA terC tia yehA yehB yehC yehD	DAEC	31	124	HOL	JUT	7	+++	Massive		30			
EC155	221	LVK	641		O70:H10	csgA fimH gad hlyE lpfA nlpI terC yehA yehB yehC yehD	NG	31	125	HOL	JUT	7	+++	Moderate	32				34
EC156	224	LVK	967		O36:H42	anr cif csgA **eae-e01-epsilon** efa1 **ehxA** espA espB espF espJ etpD fdeC fimH gad hlyE iha **iss** iucC **iutA** nleA nleB nleC nlpI **ompT stx1a-O157-FLY16 stx2a-O157-SF-258-98** terC tir traT yehA yehB yehC yehD **stx1 stx2**	NG	33	127	HOL	JUT	15	+	Moderate					

EC157	225	LVK	1049		O160/O8:H10	csgA fimH gad hlyE lpfA nlpI terC yehA yehB yehC yehD	NG	12	66	HOL	JUT	8	++	-			18		
EC158	227	LVK	1716		O130:H26	csgA fimH gad hlyE **iss** lpfA nlpI **ompT** terC yehA yehB yehC yehD	NG	65	67	HOL, MIX	JUT	7	++	Weak					
EC159	229	LVK	2325		O153:H25	AslA, anr, csgA, fdeC, fimH, hha, hlyE, nlpl, terC, traJ, traT, yehA, yehB, yehC, yehD	NG	43	106	JER	FYN	6	+++	Massive			31		
EC160	230	LVK	2524		O6:H33	AslA, **afaA, afaB, afaC, afaD**, csgA, fdeC, fimH, gad, hlyE, hra, ireA, **iroN**, iss, iucC, **iutA**, mcbA, mchB, mchC, mcmA, nlpl, **papA_F48, papC**, shiA, sitA, terC, tia, yehA, yehB, yehC, yehD	NG	43	107	JER	FYN	8	+++	Massive		34	23		
EC161	233	LVK	ST		O1/O15/O17/ O9/O9a/O8: H4/H18/ H34/H55	AslA, cea, csgA, fdeC, fimH, gad, hha, hylE, iss, iucC, **iutA**, nlpl, **ompT**, sitA, terC, traJ, traT, yehA, yehB, yehC, yehD	DAEC/ ExPEC	14	71	HOL, MIX	JUT	11	++	Massive			28		
