# Supplementary material for: Diversity of Escherichia coli from Faecal Samples of Danish Calves with Diarrhoea
Source: Vet Sci. 2025 Oct 13;12(10):987. doi: 10.3390/vetsci12100987 (PMC12568266; doi:10.3390/vetsci12100987)
Supplement: Supplementary file 1 [file vetsci-12-00987-s001.zip › Supplementary Table S4. ST-types of E. coli.pdf]

Supplemental Table S4. Sequence typing of 161 isolates of *E. coli* obtained from calves with diarrhoea.

ST (genotypes)	*E. coli*		
	with no other detected pathogens (n=30)	other detected pathogen(s) (n=69)	+ unknown presence of other pathogen(s) (clinical cases) (n=62)
10 (ETEC (n=8), NG (n=6), F5 positive (n=2), DAEC/ExPEC (n=1)	1	9	7
21 (ETEC (n=3)	0	0	3
34 (NG n=3)	2	1	0
58 (NG (n=6), ExPEC (n=6), DAEC/ExPEC (n=6)	2	9	7
69 (DAEC/ExPEC (n=9) NG (n=2), ExPEC (n=2), DAEC (n=2))	2	5	8
88 (DAEC/ExPEC (n=7) NG (n=4), ExPEC (n=2), DAEC (n=1))	6	3	5
108 (DAEC/ExPEC (n=3) NG (n=2), ExPEC (n=2), DAEC (n=1), DAEC/EHEC (n=1)	1	4	2
117 (DAEC/ExPEC (n=3) , ExPEC (n=2), DAEC (n=2))	0	0	6
155 (DAEC/ExPEC (n=1) , NG (n=2)	1	0	2
219 (DAEC/EHEC (n=2) , NG (n=1)	1	1	1
362 (DAEC/ExPEC (n=6)	1	5	0
1049 (NG (n=2), ExPEC (n=1))	1	1	1
**STs occurring only once or twice**			
17,23, 29, 32, 38, 56, 109, 120, 164, 167, 174, 187 ,223, 227, 301, 398, 351, 410, 540, 602, 611, 641, 744, 939, 967, 1485, 1725, 2325, 2522,2524, 3042, 3995, 5177, 5236, 5911 ,6118, 17734, 5880	7	10	12

For 99 of the isolates the faecal samples were assessed for the presence of diarrhoea-associated pathogens other than *E. coli*, while 62 isolates originated from calves with presumed *E. coli* diarrhoea (without investigation of potential simultaneously presence of other pathogens).
