# Supplementary material for: Diversity of Escherichia coli from Faecal Samples of Danish Calves with Diarrhoea
Source: Vet Sci. 2025 Oct 13;12(10):987. doi: 10.3390/vetsci12100987 (PMC12568266; doi:10.3390/vetsci12100987)
Supplement: Supplementary file 1 [file vetsci-12-00987-s001.zip › Supplementary Table S5. Serotypes of E. coli detected in calves with diarrhoea.pdf]

Supplemental Table S5. Serotyping of 161 isolates of *E. coli* obtained from calves with diarrhoea.

Serotypes		*E. coli*			
O	H	n	with no other detected pathogens (n=30)	other detected pathogen(s) (n=69)	+ unknown presence of other pathogen(s) (clinical cases) (n=62)
8	21	1	1	0	0
	20	1	1	0	0
	9	3	2	0	1
	25	3	1	2	0
	10	3	0	2	1
	17	2	0	1	1
	4	1	0	0	1
	-	1	0	0	1
9	9	2	0	2	0
	10	1	0	0	1
	21	1	0	0	1
	25	1	1	0	0
	-	1	0	0	1
13	11	1	1	0	0
	15	1	1	0	0
15	18	4	2	2	2
	4	1	0	1	0
	6	1	0	1	0
	-	4	0	0	4
25	8	1	1	0	0
	28	3	0	1	2
26	11	7	1	3	3
	4	0	0	0	1
	-	0	0	0	1
45	25	1	0	0	1
	19	1	0	1	0
	2	1	0	0	1
86	25	2	1	1	0
	19	2	0	0	2
88	8	1	0	1	0
	25	1	1	0	0
99a	25	2	1	1	0
99	33	2	1	1	0
101	9	16	1	7	8
	10	4	0	3	1
154	25	1	0	1	0
	30	2	0	2	0

160	10	2	1	1	0
**O-Serotypes occurring only once or twice (n=77)**					
O91:H7, O6:H33, O6:H25, O36:H42, O130:H26, O107:H30, O107:H54, O110:H2, O132:H18, O153/-, O153:H25, O176:H30, O18ac:H17, O17:H18, O32:H9, O3:H9, O50:H25, O109:H45, O117:H16, O117:H10, O23:H31, O35:H21, -/H37,-/H27, O7:H4, O121:H5, O121:H10, O150:H8, O150:H7, O103:H2, O21:H9, O83:H42, O70:H10, O160/:H10, O153:H25, O6:H33, -/H21, O13/:H11, O133:H20, -/H15, O149:-, O149:-, -/H51, O28:H8, 070:-, O40:H32, O33:H4, -:/H7, O119:H4, -/H4, -/H6, O80:H2, O96:H4					

For 99 of the isolates the faecal samples were assessed for the presence of diarrhoea-associated pathogens other than *E. coli*, while 62 isolates originated from calves with presumed *E. coli* diarrhoea (without investigation of potential simultaneously presence of other pathogens).
